# A comparative study of structures and structural transitions of secondary transporters with the LeuT fold

**DOI:** 10.1007/s00249-012-0802-z

**Published:** 2012-05-03

**Authors:** Gunnar Jeschke

**Affiliations:** Laboratory of Physical Chemistry, ETH Zürich, Wolfgang-Pauli-Strasse 10, 8093 Zürich, Switzerland

**Keywords:** Membrane proteins, Secondary active transport, Elastic network models, Protein structure, Protein dynamics

## Abstract

Secondary active transporters from several protein families share a core of two five-helix inverted repeats that has become known as the LeuT fold. The known high-resolution protein structures with this fold were analyzed by structural superposition of the core transmembrane domains (TMDs). Three angle parameters derived from the mean TMD axes correlate with accessibility of the central binding site from the outside or inside. Structural transitions between distinct conformations were analyzed for four proteins in terms of changes in relative TMD arrangement and in internal conformation of TMDs. Collectively moving groups of TMDs were found to be correlated in the covariance matrix of elastic network models. The main features of the structural transitions can be reproduced with the 5 % slowest normal modes of anisotropic elastic network models. These results support the rocking bundle model for the major conformational change between the outward- and inward-facing states of the protein and point to an important role for the independently moving last TMDs of each repeat in occluding access to the central binding site. Occlusion is also supported by flexing of some individual TMDs in the collectively moving bundle and hash motifs.

**Electronic supplementary material:**

The online version of this article (doi:10.1007/s00249-012-0802-z) contains supplementary material, which is available to authorized users.

## Introduction

Transport of substrates across biological membranes against a concentration gradient is required for cell metabolism and signaling between cells. In secondary active transport a membrane protein couples this energetically unfavorable substrate translocation to energetically favorable translocation of an ion or several ions along an electrochemical gradient. The substrate and ion(s) may move in the same direction (symport) or opposite direction (antiport). In both cases transport is thought to conform to the alternating access model (Jardetzky 1966). This model stipulates that a central substrate binding site in the protein is accessible either from the outside or from the inside of the membrane. Detailed understanding of the transport process then depends on characterization of the structural transitions involved in substrate and ion binding and, in particular, in the switch from the outward-facing to the inward-facing states (Klingenberg 2006).

For decades, such detailed understanding was hampered by a lack of high-resolution structures of any secondary active transporter. With advances in crystal structure determination this situation has changed (Abramson et al. 2003; Huang et al. 2003). This is particularly true for the LeuT fold, which was first observed for a bacterial amino acid transporter that is a homolog of eukaryotic neurotransmitters (Yamashita et al. 2005, Zhou et al. 2007; Singh et al. 2008). The main characteristic of the LeuT fold is the occurrence of two five-helix inverted repeats that include the substrate and ion translocation channel. Such a ten-helix core consisting of two inverted repeats was later found in sodium/substrate symporters (Faham et al. 2008; Weyand et al. 2008; Ressl et al. 2009), a cooperative substrate/product antiporter (Schulze et al. 2010; Tang et al. 2010), and proton/substrate antiporters (Fang et al. 2009; Shaffer et al. 2009; Gao et al. 2010; Kowalczyk et al. 2011) from different protein families, despite insignificant sequence homology between proteins that share this fold. Indeed, sequence homology is weak even between the two inverted repeats of a given protein.

Based on these crystal structures for the LeuT fold a number of modeling studies were performed (Forrest et al. 2008; Watanabe et al. 2010; Zhao et al. 2010; Shi and Weinstein 2010; Adelman et al. 2011, Koldsø et al. 2011), and the transport mechanism was discussed in general (Abramson and Wright 2009; Forrest et al. 2011, Rudnick 2011).

However, as the crystal structures are static snapshots of a dynamic process, they do not imply a unique mechanistic interpretation. Crystal structures do not exist for all conformations that are relevant during the transport process in any of the secondary transporters. Furthermore, crystallization often requires harsh detergents or physiologically unrealistic substrate concentrations. This may lead to stabilization of conformations that are off-path with respect to substrate and ion translocation (Quick et al. 2009; Cross et al. 2011; Mchaourab et al. 2011). Perhaps not surprisingly, this has led to a controversy about the main movement between the outward-facing and inward-facing conformations, with a rocker-switch rigid-body motion of a bundle of helices (Forrest et al. 2008) and systematic changes in the tilt angles of partially unwound helices (Yamashita et al. 2005; Zhao et al. 2010, 2011) being possible contenders. A recent simulation study on a homology model for the related human serotonin transporter indicates that both types of motions contribute (Koldsø et al. 2011).

In this situation, further experimental information on the structural changes is required. Such information can be obtained on secondary transporters in environments that are closer to biological membranes by probe techniques, such as fluorescence resonance energy transfer (Zhao et al. 2010) and electron paramagnetic resonance (EPR) spectroscopy (Jeschke et al. 2004; Hilger et al. 2005, 2007, 2009; Smirnova et al. 2007; Claxton et al. 2010). With such techniques structural changes can be observed on adding ions or substrate. Pulse EPR measurements of distance distributions (Jeschke and Polyhach 2007) can reveal that some functional states are broad ensembles of structures while others are better defined (Claxton et al. 2010; Mchaourab et al. 2011).

Conclusions from probe techniques are mainly limited by a small number of distance constraints. This lack of detailed information results from the fact that each constraint requires construction, labeling, and measurement of an individual mutant protein. Therefore, pairs of labeling sites need to be selected very carefully, which may require hypotheses on the expected structural transitions. Although discussions of the transport mechanism in the LeuT fold exist (Abramson and Wright 2009; Forrest et al. 2011; Rudnick 2011), a systematic comparison of *all* existing structural information is still missing. Such a study can provide sets of testable predictions on distance changes induced by substrate and ion addition.

Furthermore, modeling of a structural transition from sparse distance constraints requires an approach that reduces the number of degrees of freedom. Such state space reduction can be based on the concept of essential protein dynamics, which stipulates that functionally relevant large-scale conformational changes are restricted to a few normal modes (Amadei et al. 1993). These modes are characterized by high collectivity of the motion and are associated with low vibration frequencies. Low-frequency normal modes can be predicted with reasonable precision and low computational effort from a *single* structure by coarse-grained elastic network models (ENM) (Bahar et al. 2010). For several pairs of soluble protein structures it has been demonstrated that structural transitions can be modeled reasonably well by driving a C^α^ atom ENM along a small number of periodically reoriented normal modes (Zheng and Brooks 2006). This approach uses a small number of long-range distance constraints to specify forces that act on the ENM and thus appears well suited for modeling with EPR distance constraints.

However, it is known that not all large-scale protein motions are modeled well by ENM (Yang et al. 2007). For instance, reconfiguration of interaction networks of H bonds and salt bridges during the structural transition as suggested for the dopamine transporter DAT with the LeuT fold (Shan et al. 2011) is unlikely to be captured by the coarse-grained ENM. It is thus an open question whether such an approach can be applied to secondary active transporters.

In this work we analyze structural variation within and structural transition of the ten-helix core in the LeuT fold of secondary active transporters based on the set of existing crystal structures. The article is structured as follows. We start by presenting a structural alignment of the core transmembrane domains (TMDs) of the seven proteins whose structures have been solved. Based on this alignment we characterize the variability of internal conformation and position of individual TMDs. We then go on to discuss how the classification of crystal structures proposed in (Forrest et al. 2011) relates to steps in the transport cycle. We identify sets of pairwise significantly different structures of the same protein for LeuT, Mhp1, AdiC, and vSGLT. For the structural transitions within these sets we provide phenomenological descriptions of the motion.

Then we turn to the question whether ENMs are a useful tool for secondary transporters in the LeuT fold. We show that the core architecture is reflected in the mode covariance matrix and discuss what conclusions on collective protein motion can be drawn from this matrix. Furthermore, we test how well structural changes in the LeuT fold are characterized by a limited number of low-frequency normal modes of the ENM and whether recomputation of the modes during the structural transition improves coverage of the coordinate change. Finally we discuss what picture emerges from our results on the large-scale structural changes.

## Methods

All protein visualization was performed with the open-source software package MMM, version 2011.1, which is available for free from our homepage (http://www.epr.ethz.ch/software/index). Structure superposition, computation of elastic network models and covariance matrices, and fitting were performed with MMM subroutines. Coarse-grained analysis of the structural transition in Mhp1 in terms of TMD mean axes was performed with home-written Matlab scripts, using MMM subroutines. Scripts that are not part of MMM can be obtained from the author on request.

### Structural alignment

All structure superpositions in this work were performed on the first chain reported in the PDB file if several chains of the transporter were present. Comparison of structure across different proteins requires alignment of corresponding residues. Owing to low sequence homology in the LeuT fold, such alignment cannot be achieved with standard sequence alignment tools. Instead, we opted for structural alignment of the ten core TMDs, starting from an assignment of these TMDs in the seven LeuT fold proteins with known structures given by (Schulze et al. 2010). We allowed for shifts of the TMDs by ±1 residue with respect to these assignments. In a set of 14 structures of the seven proteins (LeuT: PDB identifiers 2A65, 2QJU, 3F3A; Mhp1: 2JLN, 2JLO, 2X79; CaiT: 3HFX, 2WSW, 2WSX; AdiC: 3NCY, 3LRB; vSGLT: 3DH4; BetP: 2WIT; ApcT: 3GIA) we shifted the residue ranges that were assigned to the individual TMDs to minimize C^α^ root mean square deviation (r.m.s.d.) from the reference structure 2A65, which has the best resolution. During the process, core TMD lengths were reduced if this was necessary to keep all core residues within the ranges originally assigned for the TMDs in (Schulze et al. 2010). Thus, the alignment defines a minimal ten-helix core.

### Standard frame

For the standard frame we have chosen the *z* axis along the bundle axis, defined as a line with minimum r.m.s.d. from all C^α^ atoms of TMDs 1, 2, 6, and 7. The z axis points towards the periplasm. The midpoints of the arm TMDs 5 and 10 define a line in the *yz* plane. That way the *x* axis is approximately in the membrane plane, connecting the bundle and hash midpoints, whereas the *z* axis is approximately along the membrane normal. With the inward-open structure 3TT3 of LeuT, we found that the first step of this procedure did not provide a bundle axis perpendicular to the membrane. Given the poor electron density near the N terminus in this structure, we excluded the first two residues of TMD 1 (residues 11 and 12) from computation of the bundle axis for all LeuT structures. This exclusion did not lead to significant changes in the orientation of the bundle axis for the other LeuT structures 2A65, 3F3A, 3GJC, and 3TT1.

### Characteristic angles

Angle θ_*Β*,4_ includes the axis of TMD 4, defined as a line with minimum r.m.s.d. from all C^α^ atoms of the TMD, and the bundle axis (mean axis of TMDs 1, 2, 6, and 7, with the first two residues of TMD 1 excluded for LeuT structures). This angle characterizes relative orientation of hash and bundle. A similar, but distinct characteristic angle for this relative orientation was introduced in (Forrest and Rudnick 2009). To characterize orientation of the two TMDs that belong to neither hash nor bundle, we define angles ϕ_5_ and ϕ_10_ between the standard frame *x* axis and the projection of TMD 5 and 10 mean axis, respectively, onto the *xy* plane.

### Elastic network model and covariance matrix

We implemented an anisotropic elastic network model (ANM) as described by (Bahar et al. 2010) into our modeling software MMM. For the force constants γ_*ij*_ we assumed an *r* ^−6^ dependence on C^α^–C^α^ distance *r* (Hinsen et al. 2000). For C^α^ atoms that are direct neighbors or next neighbors in the peptide chain, we increase this force constant by a factor of 10,000 to constrain the corresponding distances, which are fixed by peptide bond geometry. A force-field-based parametrization came to a similar result for direct neighbors (Hinsen et al. 2000), while the necessity to constrain the next neighbor distance was recognized by (Zheng and Brooks 2006), who implemented this constraint in a different way.

Diagonalization of the Hessian matrix of the ANM provides a matrix of eigenvectors ***u***, corresponding to the normal modes of the ANM. For a model with *n* C^α^ atoms, the 3*n* eigenvectors take the form

1 $$ {\user2{u}}_{k} = (\Updelta x_{1}, \Updelta y_{1}, \Updelta z_{1}, \Updelta x_{2}, \Updelta y_{2}, \Updelta z_{2}, \ldots, \Updelta x_{n}, \Updelta y_{n}, \Updelta z_{n} ) $$

where the Δ*x*_*i*_ are displacements of the *x* coordinate of the C^α^ atom with number *i*. From the set of eigenvectors, a per-residue covariance matrix ***C*** can be computed by rewriting ***u***_*k*_ as

2 $$ {\user2{u}}_{k} = (\Updelta {\user2{r}}_{k,1} ,\Updelta {\user2{r}}_{k,2} , \ldots ,\Updelta {\user2{r}}_{k,n} ) $$

where the Δ***r***_*k,i*_ are Cartesian displacement vectors in mode *k* for atom *i*. The matrix elements *C*_*ij*_ of ***C*** are given by

3 $$ C_{ij} = \sum\limits_{k = 7}^{3n} {\frac{{\Updelta \user2{r}_{k,i} \cdot \Updelta \user2{r}_{k,j} }}{{\lambda_{k} }}} $$

where the λ_*k*_ are eigenvalues of the Hessian matrix. The eigenvectors are ordered by ascending eigenvalue, and the first six eigenvectors are neglected, as they correspond to overall rotation and translation of the peptide chain.

### Coverage of conformational changes by slow modes of an ANM

The coordinate change between two structures can be written as a vector Δ***R*** that is structured in the same way as the ***u***_*k*_ in Eq. (1). With the complete matrix ***u*** of eigenvectors of the Hessian, the system of linear equations

4 $$ \Updelta {\user2{R}} = {\user2{ud}} $$

has a unique solution for the coefficient vector ***d***. In other words, the coordinate change Δ***R*** can be expressed as a linear combination of displacements along the normal modes of the ANM that are represented by the eigenvectors. If the two structures have been superimposed before by finding the rotation and translation that minimize the C^α^ r.m.s.d., the first six coefficients *d*_1_… *d*_6_ are exactly zero. In this case, the basis can be reduced by excluding eigenvectors ***u***_1_… ***u***_6_. Furthermore, all coefficients *d*_*k*_ can be taken as positive, as multiplication of an eigenvector ***u***_*k*_ with −1 provides another valid set of normal modes.

With a given energy, larger displacements can be obtained along normal modes with low eigenvalues, which correspond to the eigenvectors ***u***_*k*_ with small *k*. It follows that the coefficients *d*_*k*_ should have a tendency to decrease with increasing index *k*.

We now consider a restricted basis ***v*** of normal modes that is constructed from the modes *k* = 7… *B* + 6 of ***u*** with *B* < 3*n* − 6. In general, a linear combination of the *B* modes with new coefficients *d*_*k*_ will not exactly reproduce the coordinate change Δ***R***. We can still solve for the coefficient vector ***d*** in a least-squares sense,

5 $$ {\user2{d}}_{\text{LSQ}} = \min_{\user2{d}} \left( {\left\| {\user2{vd}} - \Updelta {\user2{R}} \right\|^{2} } \right) .$$

The remaining r.m.s.d. Δ_*B*,0_ between ***vd*** and Δ***R*** can be compared to the C^α^ r.m.s.d. between the two structures Δ_exp_ in order to assess completeness of the reduced basis ***v*** in describing the structural transition. The ratio *f*_0_ = (Δ_exp_ − Δ_*B*,0_)/Δ_exp_ is a measure for the fraction of the structural change that is covered by the reduced basis of normal modes. Reduction of the basis of normal modes can be characterized by the fraction of modes *b* = *B*/(3*n* − 6).

The solution of Eq. (5) is not necessarily the best description of the structural change that can be obtained with an ANM with *B* modes. This is because the normal modes are computed in a harmonic approximation, which is not valid for large-scale structural changes. The problem can be reduced by scaling ***d***_LSQ_ by a factor *s* so that δ***R*** = *s·* ***vd***_LSQ_ corresponds to only a small coordinate change. Here we limit the maximum coordinate change of any C^α^ atom to 0.2 Å. After adding δ***R*** to the coordinates, we compute a new Hessian and new normal modes and solve Eq. (5) again to obtain a new set of coefficients ***d***_LSQ_ for the next step. This procedure is then iterated until the coordinate set converges.

In all cases except one this algorithm converged to a final coordinate set ***R***_f_ after less than 150 iterations. We observed very slow convergence for the transition 3OB6 → 3L1L and stopped the fitting after 400 iterations. Owing to the basis reduction, the final coordinate set ***R***_f_ differs from the coordinates of the experimental end point structure. We denote the r.m.s.d. between ***R***_f_ and the experimental end point structure as Δ_*B*,1_. The fractional coverage *f*_1_ = (Δ_exp_ − Δ_*B*,1_)/Δ_exp_ is a measure for the fraction of the structural change that is covered by the reduced basis of continuously updated normal modes. This procedure is similar in spirit to the algorithm of (Zheng and Brooks 2006), except that we drive the transition directly to the known endpoint structure instead of relying on a small number of distance constraints. This simpler problem does not suffer from potential overfitting.

## Results and discussion

### Structural alignment

The core alignment of all transporters in the LeuT fold that have been crystallized to date is shown in Table 1. Note that TMD numbering refers to the core, whereas in some proteins additional TMDs exist N-terminally from the core, so that TMD numbering in the original publications on the structures may differ. For example, there is one additional N-terminal TMD in vSGLT; thus, the core TMD 1 is TMD 2 in the structure. In our assignment for Mhp1, TMD 9 and 10 are direct neighbors without an intervening loop. While it might be more appropriate to assign the two border residues as a short turn, our subsequent discussions are not affected by such redefinition.

**Table 1:** Alignment of core TMDs for seven secondary transporters from the LeuT fold

Protein	LeuT	Mhp1	BetP	ApcT	CaiT	AdiC	vSGLT
TMD 1	11–35	29–53	138–162	10–34	88–112	11–35	53–77
TMD 2	42–67	59–84	180–205	40–65	133–158	41–66	82–107
TMD 3	89–120	102–133	234–265	85–116	188–219	81–112	126–157
TMD 4	168–183	142–157	280–295	125–140	232–247	125–140	162–177
TMD 5	191–214	164–187	302–325	147–170	255–278	144–167	185–208
TMD 6	240–264	206–230	359–383	186–210	309–333	190–214	249–273
TMD 7	275–300	249–274	397–422	221–246	347–372	224–249	282–307
TMD 8	336–367	294–325	449–480	268–299	403–434	274–305	346–377
TMD 9	379–394	340–355	492–507	321–336	449–464	327–342	399–414
TMD 10	401–424	356–379	515–538	340–363	469–492	351–374	424–447
PDB^a^	2A65	2JLN	2WIT	3GIA	3HFX	3NCY	3DH4
C^α^ r.m.s.d. (Å)^b^	0.0	3.28	3.71	3.83	3.87	4.02	4.43

^a^Entry of a representative structure

^b^With respect to LeuT structure 2A65. For proteins with several structures, the number corresponds to the representative structure

**Scheme 1:**
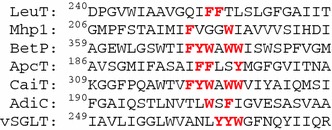
Sequence alignment for core TMD 6

The total core size is 241 TMD residues. The list of lengths of the ten TMDs (27, 23, 32, 17, 26// 22, 22, 31, 17, 24) reflects pseudosymmetry of the two inverted repeats, which are separated here by the double slash. Although sequence homology in the LeuT fold is generally poor, we checked for any peculiarities in the sequence alignments of the individual TMDs. The only distinctive feature is a high incidence of aromatic residues near the region of TMD 6, which is unwound in most, but not all structures in the LeuT fold (Scheme 1). This feature may be functionally relevant. Of these residues F253 in LeuT has been implied in occlusion of the periplasmic pathway to the central binding side and Y263 in vSGLT in occlusion of the cytoplasmic pathway (Abramson and Wright 2009). Furthermore, by molecular dynamics (MD) simulations rotamer changes of residues from this range were implied in changes of accessibility of the central binding site for the substrate for both LeuT (Claxton et al. 2010) and the dopamine transporter DAT, which shares the LeuT fold (Shan et al. 2011). No high incidence of aromatic residues is found near the unwound region of the pseudosymmetry-related TMD 1 of the first repeat.

**Fig. 1:**
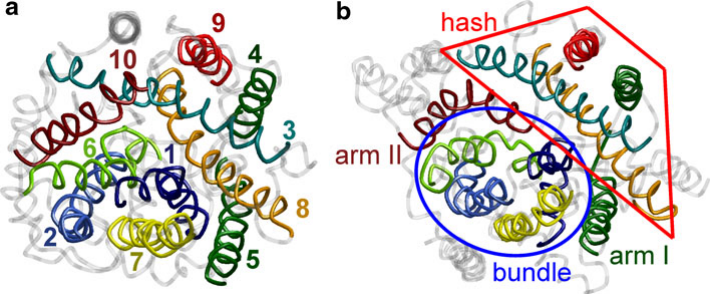
Core architecture of LeuT structure 2A65 (**a**) and vSGLT structure 3DH4 (**b**). TMDs are color coded with numbering starting with the first core TMD. Collectively moving groups of TMDs are marked as bundle and hash motif in (**b**)

The similarity of the core architectures can be appreciated from Fig. 1, where the outward occluded structure of LeuT and the structure most distant from it (maximum core C^α^ r.m.s.d.) are shown from a view that is approximately perpendicular to the membrane.

The C^α^ r.m.s.d. for pairwise alignment of the cores of different proteins in the LeuT fold varies between 1.86 (CaiT: 3HFX and BetP: 2WIT, both from the BCCT family) and 5.87 Å (vSGLT: 3DH4 and AdiC: 3NCY). This can be compared to the structural change between the outward-open structure of Mhp1 (2JLN) and the inward-open structure of the same protein (2X79), which is 2.93 Å. The comparison suggests that the differences of core TMD internal conformation and orientation between the individual proteins are not exclusively due to idiosyncrasies of the proteins, but may be related at least partially to different conformational states of the shared architecture.

**Fig. 2:**
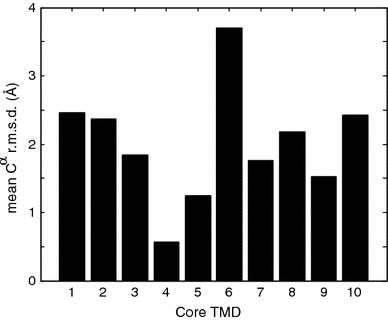
Conformational variability of core TMDs in the LeuT fold characterized by the mean r.m.s.d. for pairwise superposition of the C^α^ atoms of each individual TMD in the structure space with PDB identifiers 2WSW, 2WIT, 3DH4, 2A65, 2JLN, 3NCY, and 3GIA

To analyze internal conformation variability of individual TMDs we have considered the mean C^α^ r.m.s.d. for pairwise superposition of single TMDs in the set of structures 2WSW, 2WIT, 3DH4, 2A65, 2JLN, 3NCY, and 3GIA (Fig. 2). For most TMDs we find a mean C^α^ r.m.s.d. between 1.5 and 2.5 Å. The exceptions are TMD 4 with a very low internal conformation variability of only 0.57 Å and TMD 6 with a very large internal conformation variability of 3.70 Å. Slightly enhanced internal conformation variability between 2 and 2.5 Å is observed for TMDs 1, 2, 8, and 10. Based on FRET measurements and steered MD simulations, TMD 1 has been implied in the transition between the outward-facing and inward-facing state of LeuT (Zhao et al. 2010), a prediction that was later confirmed by a crystal structure for an inward-facing state (Krishnamurthy and Gouaux 2012).

Comparison of the outward occluded structure of LeuT (2A65), an occluded structure of BetP (2W8A, meanwhile superseded by 2WIT), and the inward-open structure of vSGLT (3DH4) suggested that TMD 8 changes from a kinked internal conformation via a slightly kinked internal conformation to a straight TMD in the outward-open to inward-open transition (Ressl et al. 2009). For the apo form of prolin/sodium symporter PutP, a helix-loop-helix model based on EPR data revealed a kink of TMD 8 very similar to the one seen in LeuT (Hilger et al. 2009), whereas the same TMD is straight in inward-open conformations of vSGLT, which belongs to the same protein family of sodium substrate symporters (SSS) as PutP. However, in Mhp1 and LeuT, which could be crystallized in outward-open and inward-open structures, only slight straightening is observed (*vide infra*). In the SLC 7 family of amino acid transporters TMD 8 is straight in the outward-open structure of the arginine/agmatine antiporter AdiC (3NCY) and kinked in the inward-open structure of ApcT (3GIA). Hence, even if a change of internal conformation of TMD 8 is a universal feature of the transition from the outward-open to the inward-open state, extent and direction of this change differ between different proteins for currently unknown reasons.

We have taken advantage of the small internal conformation variability of TMD 4 to define a robust parameter θ_*Β*,4_ (see Sect. “Methods”) that characterizes relative orientation of the bundle of TMDs 1, 2, 6, and 7 and the hash motif made up by TMDs 3, 4, 8, and 9 (see Fig. 1b). Such a parameter is of interest as motion of the bundle relative to scaffold TMDs (Forrest et al. 2008) or to the hash motif (Shimamura et al. 2010) was suggested as the main conformational change in the outward-open to inward-open structural transition. In structures that were previously assigned as outward-open or outward-occluded (Ce, CSe, CSec) angle θ_*Β*,4_ is larger than 26° with typical values around 30° (Table 2). The only exception is the CSec conformation of the antiporter AdiC (3L1L), which has θ_*Β*,4_ = 19.2°. For all inward-open structures, we find θ_*Β*,4_ < 18° with typical values around 15°. The BetP structure 2WIT, which is assigned as CSic, but was originally assigned as CSc, has θ_*Β*,4_ = 17.4°.

**Table 2:** Coarse-grained characteristics of conformations in the LeuT fold

PDB	3HFX	2WSX	2WIT	3DH4	2XQ2	3TT1	3F3A	2A65	3GJC
Protein	CaiT	CaiT	BetP	vSGLT	vSGLT	LeuT^a^	LeuT^a^	LeuT^a^	LeuT^a^
Conformation	CSi	CSi	CSic	CSic	Ci	Ce	CSe	CSec	S2
θ_*B*,4_ (°)	16.1	16.3	17.4	11.5	17.7	33.3	33.0	29.0	29.9
ϕ_5_ (°)	12.9	15	27.7	9.5	2.1	38.5	38.2	33.1	30.6
ϕ_10_ (°)	30.1	28.1	24.8	45.6	48.3	10.9	10.8	15.8	14.1

PDB	3TT3	2JLN	2JLO	2X79	3GIA	3LRB	3OB6	3L1L
Protein	LeuT^a^	Mhp1	Mhp1	Mhp1	ApcT	AdiC	AdiC	AdiC
Conformation	Ci	Ce	CSec	Ci	Cic	Ce	CSe	CSec
θ_*B*,4_ (°)	12.5	29.3	26.7	9.6	16.2	26.7	28.2	19.2
ϕ_5_ (°)	0.1	33.5	29.3	5.2	5.7	21.5	18.0	19.9
ϕ_10_ (°)	25.6	11.8	30.7	48.3	26.7	15.2	16.1	25.1

Conformation assignments were taken from (Forrest et al. 2011), except for 2XQ2, 3TT1, 3TT3, and 3OB6, which were assigned analogously. S2 is an outward-open structure with a putative secondary binding site blocked

^a^For LeuT, the first two residues of TMD1 were excluded from computation of the bundle axis

### Relation between crystalline conformations and functional states

**Scheme 2:**
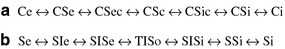
Secondary transporter conformations (**a**) and functional states (**b**)

Recently a classification was suggested for secondary active transporter conformations encountered in crystal structures (Forrest et al. 2011). In this classification (Scheme 2a) the outward-open to inward-open transition starts from the Ce conformation, where no substrate or ions are bound, and the central binding site is fully accessible from the outside and inaccessible from the inside. It proceeds via the CSe conformation, where substrate and ion(s) are bound and the central binding site is still fully accessible from the outside to the CSec conformation, where the central binding site becomes weakly occluded. The pivot point of the transition is the CSc conformation where the central binding site is strongly occluded with respect to both the outside and inside. This conformation then converts to CSic, where the binding site is only weakly occluded to the inside, but inaccessible from the outside. From the inside-open conformation CSi substrate and ion(s) can dissociate to give the inside-open apo conformation Ci. In symporters, Ci must be able to convert back to Ce by thermal excitation to close the cycle, whereas in antiporters this conversion must be forbidden to avoid uncoupling of substrate and ion transport.

From a functional point of view the succession of states is slightly different. To minimize uncoupled transport, in symporters the ion must bind to the transporter before the substrate (“first on”) and must also unbind before the substrate (“first off”) (Forrest et al. 2011). We neglect intermediate transition states, except for the occluded state TISo where the central binding site is inaccessible from both the outside and inside. In this picture (Scheme 2b) the outward-open apo state Se first binds the ion to give SIe and then the substrate to give SISe. This state converts via the transition state TISo to an inward-open, substrate and ion-bound state SISi, which first loses the ion to give SSi and then the substrate to give the inward-open apoprotein Si.

As pointed out by (Mchaourab et al. 2011) and as is known from NMR studies on soluble enzymes (Henzler-Wildman and Kern 2007), functional states are not necessarily associated with well-defined conformations. Such states may correspond to ensembles of conformations, and state changes may be associated with shifting weights between distinct subensembles. Despite this complication, structural changes between the conformations indicated in Scheme 2a are of interest for understanding the functional states. These changes reveal which parts of the structure are flexible and which parts move collectively. This is still true for conformations that may be off-path because of blocking of a binding site by detergent, as suggested for structure 2A65 of LeuT (Quick et al. 2009) but dismissed by (Wang et al. 2012), or downregulated because of low osmolarity, as suggested for structure 2WIT of BetP (Forrest et al. 2011). In the following, we thus turn to an analysis of structural changes.

### Identification of distinct protein conformations in the LeuT fold

For identification of structural transitions we originally considered all protein structures in the LeuT fold that were published in the PDB by the end of 2011. Of the 23 structures of LeuT bound to different substrates and inhibitors, 18 structures are within 0.3 Å C^α^ r.m.s.d. from the best resolved structure 2A65. Structures 3QS5 and 3QS6 differ by only 0.45 and 0.5 Å from 2A65 and by only 0.26 Å from each other. This group of 20 structures was assigned to the CSec conformation. For the remaining three structures 3F3A (1.20 Å C^α^ r.m.s.d.), 3GJC (1.99 Å), and 3QS4 (1.20 Å), we computed pairwise C^α^ r.m.s.d. of the core, including TMDs and intervening loops. This revealed that structure 3F3A with the competitive inhibitor tryptophan bound in a second binding site and structure 3QS4 of mutant F259V also bound to tryptophan agree within 0.24 Å. These structures represent a CSe conformation. Structure 3GJC of mutant E290S with *n*-octyl-β-d-glucopyranoside (OG) bound in the same site deviates by 1.10 Å from these structures and was assigned as a conformation with blocked secondary binding site S2 by (Forrest et al. 2011).

While this work was under review, a number of new LeuT structures appeared. The first group of these structures addressed the problem of possible OG binding and other influence of detergents in the structures previously assigned to the CSec state by growing crystals from bicelles and using a selenium-containing analog of OG (Wang et al. 2012). In the second work, rationally designed mutants and complexation with antibody fragments provided, among others, the inward-open structure 3TT3 (Ci state) and an outward-open substrate-free structure 3TT1 (Ce state) (Krishnamurthy and Gouaux 2012). The core of structure 3TT1 deviates by only 0.42 and 0.50 Å from the core of structures 3QS4 and 3F3A (CSe state), respectively, a fact already noticed by Krishnamurthy and Gouaux. Structure 3TT3 deviates by 3.70, 3.34, and 3.16 Å from 3F3A, 3GJC, and 2A65, respectively. For all the remaining new LeuT structures the core deviation from 2A65 (CSec state) does not exceed 0.49 Å. All structures obtained by crystallization from bicelles agree better with each other (maximum deviation 0.18 Å) than with structure 2A65 (typical deviation 0.48 Å), except for structure 3USI. For LeuT we shall thus consider structural transitions between 3TT1, 3F3A, 2A65, 3GJC, and 3TT3, which are the best resolved structures in their respective groups.

The three Mhp1 structures 2JLN, 2JLO, and 2X79 are all distinct with C^α^ r.m.s.d. of 1.13 Å between 2JLN and 2JLO and 3.03 Å between 2JLO and 2X79. They combine to a succession of conformations Ce ↔ CSec ↔ Ci.

The two BetP structures 2WIT with substrate glycine betaine bound and 3P03 of mutant G153D with choline bound have a core C^α^ r.m.s.d. of 1.04 Å. The significant deviations are confined to the loops between TMDs. As loop structure is easily influenced by crystal packing, we refrain from further study of the difference between these two structures.

The three ApcT structures 3GI8, 3GI9, and 3GIA have mutual C^α^ r.m.s.d. of less than 0.5 Å, which is insignificant at their resolution. The two CaiT structures 3HFX and 2WSX of the same protein differ by 1.43 Å. However, as in the case of BetP, significant differences are strictly confined to the loops. Hence, we also refrain from discussing structural changes in ApcT and CaiT.

In contrast, the five AdiC structures 3LRB (apo), 3LRC (apo), 3L1L (arginine bound), 3NCY (apo complexed with a Fab fragment), and 3OB6 (mutant N101A arginine bound in an open-to-outward conformation) are pairwise significantly different with C^α^ r.m.s.d. larger than 2 Å, except for the pair 3LRB/3LRC, which corresponds to the same conformation. Structures 3LRB and 3NCY can be assigned to different Ce conformations, 3OB6 to a CSe conformation, and 3L1L to a CSec conformation. We shall consider the sequence of structural transitions 3LRB ↔ 3OB6 ↔ 3L1L as well as the transition 3LRB ↔ 3NCY.

Finally, the cores of the galactose-bound structure (3DH4) and the apo structure (2XQ2) of vSGLT differ by 2.19 Å. These structures can be assigned as CSic and Ci conformations. Note that a computational study has assigned structure 3DH4 as an ion-releasing state (Li and Tajkhorshid 2009). In the nomenclature of Scheme 2b this structure thus corresponds to an SSi rather than an SISi state.

### Coarse-grained analysis of the Ce ↔ Ci transition in Mhp1

**Fig. 3:**
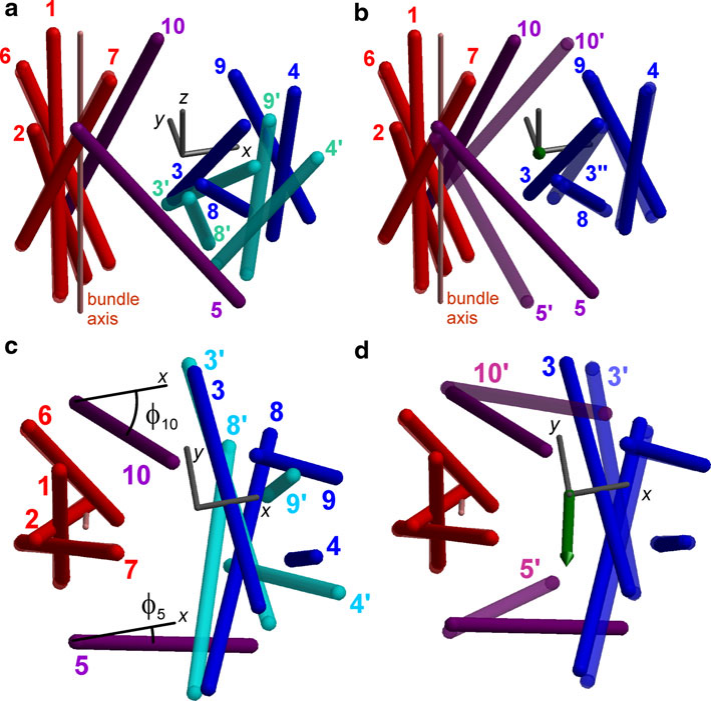
Coarse-grained visualization of the ten-helix core in Mhp1 structures 2X79 (*inward open*, *solid red*, *blue*, and *purple*) and 2JLN (*outward open*, *transparent red* and *cyan*). **a** Structures are superimposed on bundle TMDs 1, 2, 6, and 7 (C^α^ r.m.s.d. 0.58 Å). The C^α^ r.m.s.d. between the two hash motifs (*blue* and *cyan*) is 6.16 Å. Coupler TMDs 5 and 10 are not displayed for structure 2JLN. The standard frame is visualized as a *grey tripod* (see text). The screw axis for the hash motif movement points to the observer. **b** A screw transformation was applied to the hash motif of 2JLN, resulting in the position and orientation shown as transparent blue sticks. Coupler TMDs 5′ and 10′ of structure 2JLN are shown as transparent purple sticks. The transformed hash motif superimposes with C^α^ r.m.s.d. of 0.89 Å onto the one of structure 2X79 (*solid blue sticks*). **c** View from the periplasmic side corresponding to (**a**). **d** View from the periplasmic side corresponding to (**b**). The screw axis is visualized by a *green arrow*

The only protein for which both an outward-open (2JLN) and inward-open structure (2X79) was known on initial submission of this work was Mhp1. The transition has been characterized as mainly a relative motion of the hash motif (TMDs 3, 4, 8, and 9) with respect to the bundle (TMDs 1, 2, 6, and 7), accompanied by some bending and flexing of the arm TMDs 5 and 10 (Shimamura et al. 2010). To characterize this movement we have superimposed the bundle TMDs by minimization of C^α^ r.m.s.d. (0.58 Å). After such superposition the C^α^ r.m.s.d. of the hash motif is 6.16 Å. The difference in orientation of the hash motif can be appreciated from the visualization in the standard frame in Fig. 3a, where TMDs are represented as sticks oriented along the mean axis of the C^α^ atoms.

Optimum superposition of the hash motif from the outward-open structure 2JLN to the inward-open structure 2X79 can be achieved by a screw transformation (minimum C^α^ r.m.s.d. 0.89 Å). A unit vector along the screw axis has polar angles θ = 52.1° and ϕ = 77.6° in the standard frame (green arrow in Fig. 3b, d). The screw transformation is a rotation by 31.5° about this axis, followed by a 1.9 Å translation along the axis. As is apparent from Fig. 3a, the remaining deviation after this screw transformation is largely confined to TMD 3.

In the coarse-grained representation, where each TMD is represented by only its mean axis, movement of the arms can be considered as a rotation about an axis intersecting the TMD midpoints. Arm I (TMD 5) rotates by 37.7° about an axis that includes an angle of 67.6° with the screw axis of the hash motif, whereas arm II (TMD 10) rotates by 19.5° about an axis that includes an angle of 39.5° with the screw axis. The view along the screw axis (Fig. 3a, b) shows that this rotation partially follows the hash rotation. The view from the periplasmic side along the membrane normal (Fig. 3d) demonstrates that the arms rotate more strongly than the hash about the membrane normal. Such movement may help to occlude one side of the substrate translocation pathway while opening up the other side.

If the same analysis is applied to the Ce ↔ CSec transition between structures 2JLN and 2JLO, only arm II rotates by 14.9°. The screw transformation of the hash and any internal changes in the bundle and hash are insignificant in this transition. Note however that structure 2JLO is based on 2JLN with remodeling of only part of loop L9–10 and TMD 10 (Weyand et al. 2008).

We now discuss reorientation of the arms characterized by angles ϕ_5_ and ϕ_10_ (Fig. 3c). We have checked that this motion is generally better described as reorientation of the corresponding TMD as a whole than as an independent movement of one moiety of a kinked TMD, in agreement with the finding that the entire TMD 5 contributes to the cyoplasmic thin gate and the entire TMD 10 to the periplasmic thick gate (Shimamura et al. 2010). Note however that a small contribution from a change in kink angle may be contained in ϕ_5_ and ϕ_10_. For inward-open conformations we find ϕ_5_ < 15°, for outward-open conformations of symporters ϕ_5_ > 26° with larger values for less occluded states. The conformation of BetP assigned either as CSic or CSc (2WIT) has ϕ_5_ = 27.7°. Outward-open conformations Ce, CSe, and CSec of the antiporter AdiC have ϕ_5_ ≈ 20°. The situation is somewhat less clear cut with respect to ϕ_10_. For most outward-open conformations we find ϕ_10_ < 18°, and for all inward-open conformations we find ϕ_10_ > 25°, with typical values of 35°. The conformation of BetP in between CSic and CSc (2WIT) has ϕ_10_ = 24.8°. However, the outward-open CSec conformation of Mhp1 (2JLO) has ϕ_10_ = 30.7°, which suggests that ϕ_10_ is more strongly correlated to occlusion of the extracellular path rather than to opening of the intracellular path. The CSec conformation of AdiC (3L1L) has ϕ_10_ = 25.1°, again suggesting that a change in ϕ_10_ (reorientation of TMD 10) is mainly related to occlusion of the periplasmic pathway and uncoupled from changes in ϕ_5_.

### Phenomenological analysis of structural transitions

#### Mhp1

**Fig. 4:**
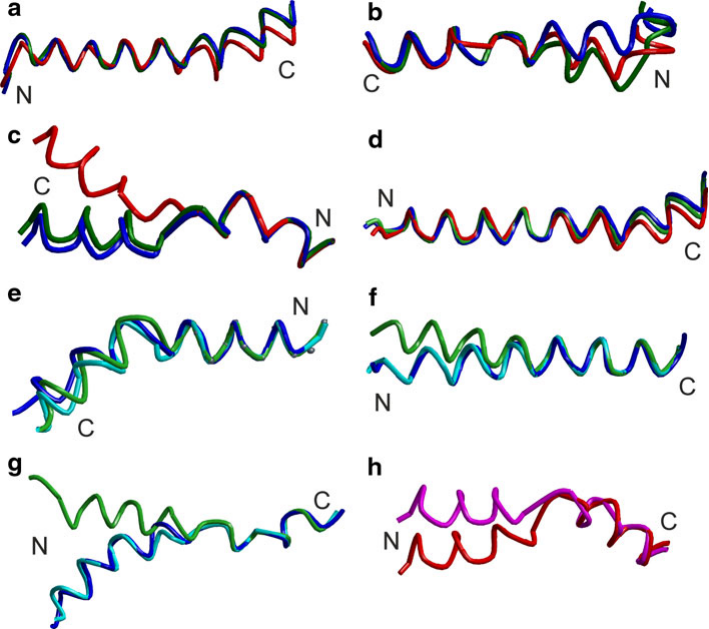
Changes of TMD internal conformations for **a** TMD 8 of Mhp1, **b** TMD 10 of Mhp1, **c** TMD 1 of LeuT, **d** TMD 8 of LeuT, **e** TMD 1 of AdiC, **f** TMD 2 of AdiC, **g** TMD 6 of AdiC, **h** TMD 1 of vSGLT. *Color code* relates to assigned conformations: Ce *blue*, CSe *cyan*, CSec *green*, CSic *magenta*, Ci *red*. The N and C terminus of the TMDs are marked

For the transition Ce (2JLN) → CSec (2JLO) the conformation change is largely restricted to TMD 10, which kinks at the N terminal end (periplasmic side) as visualized in Fig. 4b. Complete visualization of the transition in Online Resource 1, page S 1, shows that TMD 10 kinks towards the bundle, thus occluding periplasmic access to the central binding site, as was already pointed out in (Weyand et al. 2008). Among the characteristic angles, θ_*Β*,4_ and ϕ_5_ only slightly decrease by 2.6 and 4.2°, respectively, corresponding to only 13–15 % of the total change between the Ce and Ci conformations (19.7 and 28.3°, respectively, for the structure pair 2JLN/2X79). In contrast, ϕ_10_ increases by 18.9°, which is approximately 50 % of the change between the Ce and Ci conformations. The kink or bending angle β_10_ between the mean axes through the N-terminal and C-terminal halves of TMD 10 increases by about 6°. Analogously defined angles for TMDs 1, 6, and 5 (β_1_, β_5_, β_6_) change by less than 3°.

The transition CSec (2JLO) → Ci (2X79) is strongly dominated by the reorientation of the hash motif with respect to the bundle. In addition, the kinks of TMD 8 and 10 slightly decrease (Fig. 4a, b). This straightening of TMD 8 is in line with comparison of structures of different symporters in outward-open and inward-open conformations. However, in the case of Mhp1 such straightening is not complete. Among the loops, L6–7 and L7–8 move away from the bundle axis to the outside with L7–8 simultaneously moving from the cytosol towards the membrane. The characteristic angle changes are a decrease of 17.1° for θ_Β,4_, a decrease of 24.1° for ϕ_5_, and an increase of 17.6° for ϕ_10_. Angle β_10_ increases by 8°, whereas β_1_, β_5_, and β_6_ change by less than 3°.

#### LeuT

For the Ce (3TT1) → CSe (3F3A) transition, TMD internal conformations are conserved within a C^α^ r.m.s.d. of about 0.4 Å. The smallest change of 0.12 Å is found for TMD 4 and the largest change of 0.43 Å for TMD 6. Angles θ_*Β*,4_, ϕ_5_, and ϕ_10_ do not change significantly (less than 0.5°). There are no significant changes in the kink and bending angles β_*k*_.

Slightly larger changes are observed for the CSe (3F3A) → CSec (2A65) transition, although the trend is the same. TMD internal conformations are conserved within a C^α^ r.m.s.d. of 0.6 Å. The smallest changes of 0.09 and 0.60 Å are again found for TMD 4 and 6, respectively, in agreement with findings on internal conformation variability of TMDs across different proteins in the LeuT fold. Although TMD 1 and 2 do not significantly kink, their periplasmic ends tilt somewhat away from the bundle axis towards the hash, which contributes to the occlusion of the central binding site (Online Resource 1, page S 3). This was already pointed out in (Singh et al. 2008). The kink angle β_1_ decreases by 19.4°.

For the CSec (2A65) → S2 (3GJC) transition, TMD internal conformations are even better preserved. Indeed, the two structures differ only slightly except for loop L3–4. Changes of the characteristic angles and kink or bending angles do not exceed 2.5°.

The largest changes are observed during the CSec (2A65) → Ci (3TT3) transition. Internal conformation of TMD 1 changes drastically by 1.68 Å, as visualized in Fig. 4c. Note that this change was predicted on the basis of atomistic MD simulations (Zhao and Noskov 2011). Significant conformation change is also observed for TMD 8 (0.68 Å), whereas all other TMDs maintain conformation within 0.5 Å C^α^ r.m.s.d., with TMD 4 again exhibiting the smallest change. Similarly to the CSec (2JLO) → Ci (2X79) transition of Mhp1, TMD 8 slightly straightens, but to an even smaller extent (Fig. 4d). Also in analogy to the CSec → Ci transition of Mhp1, θ_*B*,4_ decreases by 16.5° (17.1° for Mhp1). We have checked whether the change in θ_*B*,4_ is significantly affected by the conformational change of TMD 1. For that, we defined the bundle axis from only TMD 1b (residues 26–35), 2, 6, and 7. Based on this definition, angle θ_B,4_^′^ differs only slightly from angle θ_*B*,4_ and decreases by 14.4°. We conclude that the rocking bundle motion makes a significant contribution to the CSec → Ci transition of LeuT. Kink angles β_1_ and β_6_ increase by 18.5° and 6.1°, respectively.

Likewise, the decrease in ϕ_5_ by 24.1° observed in the the CSec → Ci transition of Mhp1 finds an analogy in a decrease of ϕ_5_ by 33° in the same transition in LeuT and the increase of ϕ_10_ by 17.6° in Mhp1 in an increase by 9.8° in LeuT. These parameters, which were defined before the Ci structure of LeuT was published, thus appear to describe common behavior of different proteins within the LeuT fold.

#### AdiC

In general, outward-open states of AdiC exhibit stronger variability of TMD internal conformations than outward-open states of Mhp1 and LeuT. In the Ce (3LRB) → CSe (3OB6) transition, the periplasmic (C terminal) moiety of the partially unwound TMD 1 slightly kinks to effect a motion of L1–2 away from the hash motif (Fig. 4e and Online Resource 1, page S6). This corresponds to an opening up of the path to the central binding site. The periplasmic (N terminal) end of TMD 8 may also slightly kink away from the periplasmic path to the binding site (not shown), although this slight change is probably within uncertainty of the crystal structures. Likewise, the sections of L7–8 that are resolved in both structures move away from this path. Changes of the characteristic angles do not exceed 3.5°, except for kink angle β_1_, which increases by 7.2°.

The structural changes are more dramatic for the CSe (3OB6) → CSec (3L1L) transition. TMDs 2 and 6 straighten (Fig. 4f, g), leading to a move of their N terminal ends into the periplasmic path to the central binding side (Online Resource 1, page S7). A significant relative movement of bundle and hash occurs, as indicated by a 9° decrease of angle θ_*Β*,4_. Likewise, angle ϕ_10_ increases by 9°. In this transition, TMDs bend or kink significantly. Kink angle β_6_ decreases by 16.3°, and the angles between the N- and C-terminal halves of TMDs 5 and 10 change by Δβ_5_ = −8° and Δβ_10_ = 9.2°. Differences between the two Ce conformations (3LRB and 3NCY) are mainly confined to loop regions.

#### vSGLT

The CSic (3DH4) → CSi (2XQ2) transition is manifest mainly in an outward kink of the cytoplasmic (N terminal) moiety of TMD 1, which leads to an opening of the cytoplasmic path to the central binding site (Fig. 4h). A slight internal conformation change of TMD 8 may also contribute to this opening, although this change may hardly exceed uncertainty of the x-ray structures (not shown). Angle θ_*Β*,4_ increases slightly by 6.2°, although generally an opening up of the cytoplasmic path is associated with a decrease of this angle. Angle ϕ_5_ slightly decreases and angle ϕ_10_ slightly increases, which is in line with expectations for an opening of the cytoplasmic path. The transition involves significant changes in kink or bending angles Δβ_1_ = −8.7°, Δβ_5_ = 8.5°, and Δβ_6_ = 5.5°.

### Interpretation of the ANM covariance matrix

**Fig. 5:**
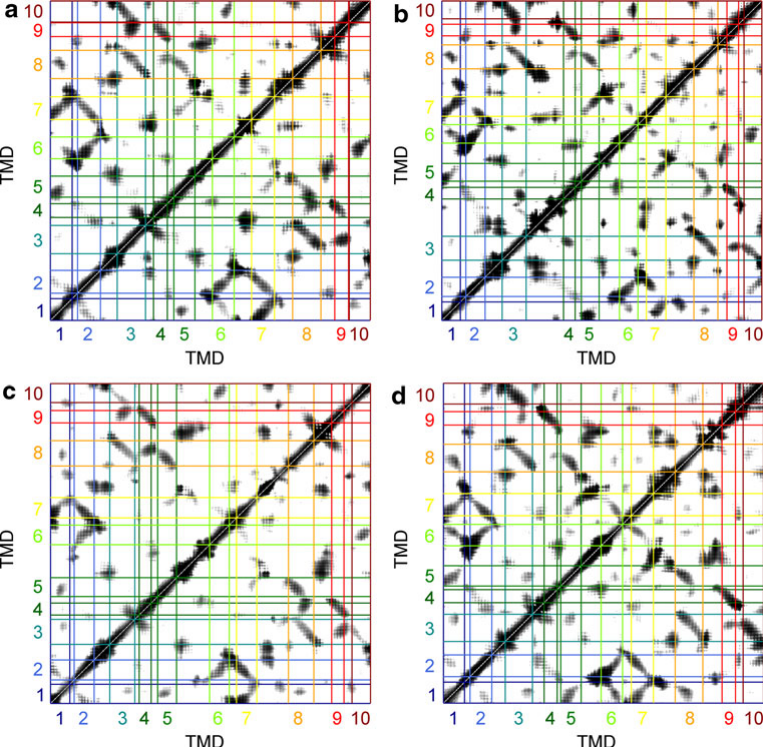
Per-residue covariance matrices of anisotropic network models for the core of proteins with the LeuT fold. **a** Mhp1 structures 2JLN in the Ce conformation (*upper left half*) and 2X79 in the Ci conformation (*lower right half*). **b** LeuT structures 3TT1in the Ce conformation (*upper left half*) and 3TT3 in the Ci conformation (*lower right half*). **c** vSGLT structure 2XQ2 in the Ci conformation. **d** AdiC structure 3OB6 in the CSe conformation

To test the predictive power and model quality of the ANM for structural transitions in the LeuT fold, we computed covariance matrices for all significantly different structures. From Fig. 5 it is clear that the common core architecture of the LeuT fold leads to common correlation features in the covariance matrix. The most obvious features correspond to collective motion within the bundle and within the hash. In the bundle, TMD 1 invariably correlates with TMD 7 and TMD 2 correlates with TMDs 6 and 7. A weaker correlation is usually seen between TMD 1 and TMD 6. These two TMDs are significantly kinked, so that their common interaction surface is reduced.

In the hash, TMD 3 correlates with TMDs 8 and 9; TMD 4 strongly correlates with TMD 9 and more weakly correlates with TMD 8. Arm I (TMD 5) couples to TMD 1 of the bundle. For outward-open states this coupling is mainly via the respective N-terminal moieties (Fig. 5a, b upper left halves and d), whereas for the inward-open states the centers of the two TMDs are more strongly coupled (Fig. 5a, b lower right halves and c). TMD 5 also couples to TMD 7 of the bundle and TMD 8 of the hash.

Likewise arm II (TMD 10) couples to both bundle and hash TMDs. The correlation of TMD 10 with TMD 6 is expected from pseudosymmetry of the inverted repeats and the analogous correlation of TMD 5 with TMD 1. Likewise, the correlation of TMD 10 with TMD 2 is analogous to the one between TMD 5 and TMD 7; however, it is much weaker for arm II than for arm I. Coupling of arm II to the hash TMD 3 is stronger in outward-open structures (Fig. 5a, b upper left halves and d) than in inward-open structures (Fig. 5a, b lower right halves and c).

Three other features are common to all covariance matrices. First, loop L3–4 exhibits correlated motion with TMDs 3 and 4, as does the pseudosymmetry-related loop L8–9 with TMDs 8 and 9. These couplings may relate to the concerted motion of the hash motif, which is composed of TMDs 3, 4, 8, and 9. Second, according to the covariance matrix the periplasmic loops L3–4 and L6–7 exhibit some kind of correlated motion. Since these loops are distant from each other, this correlation must arise from highly collective modes, such as the modes that correspond to the relative movement of hash and bundle. Third, the only direct coupling between a hash and bundle TMD involves TMDs 1 and 8, and is moderate in outward-open structures and weak or absent in inward-open structures. As pointed out by a reviewer, TMD 1 and 8 form the conserved sodium-binding site Na 2 (Abramson and Wright 2009; Zhao and Noskov 2011). The change in coupling of these two TMDs could thus be related to sodium drawing them together to stabilize the open-out conformation (Zhao and Noskov 2011). Indeed, a new LeuT structure shows that a move of the periplasmic moiety of the partially unwound helix TMD 1 is coupled to release of the sodium ion and opening of the intracellular gate (Krishnamurthy and Gouaux 2012). Such motion may then well be related to the relative motion between hash and bundle. We have checked that all these features are also observable in the covariance matrices of the other structures discussed in this work (data not shown).

The preference for couplings within the hash and bundle motifs over couplings between these motifs supports the assumption of relatively rigid, independently moving hash and bundle domains. A similar observation was made with coarse-grained Gō models (Adelman et al. 2011).

### Coverage of the structural transitions by a reduced set of ENM modes

ENM can be used to characterize conformational changes of proteins with a small number of distance constraints (Zheng and Brooks 2006). Such an approach would be of interest for characterizing states of transporters, which could not yet be crystallized, by site-directed spin labeling and EPR distance measurements. However, as previous tests of the approach have been performed only for soluble proteins and almost exclusively for interdomain hinge motion, it is not clear whether ENM can also cover the conformational changes of transporters. We shall test this hypothesis in the following.

**Fig. 6:**
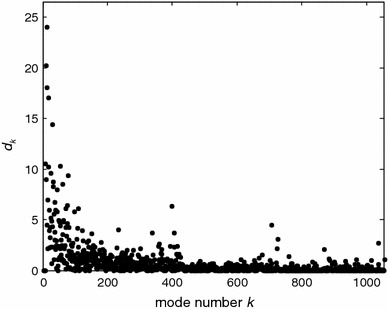
Dependence of normal mode coefficients *d*_*k*_ on mode number *k* for expressing the coordinate change between Mhp1 structures 2JLN and 2X79 as a linear combination of ANM normal modes

As demonstrated in Fig. 6 for the example of the Ce → Ci transition of Mhp1, the tendency for mode coefficients *d*_*k*_ to decrease with increasing index *k* is indeed observed for structural changes in the LeuT fold. In other words, slow modes of the ANM cover a substantial part of the conformational changes. However, some of the coefficients with higher numbers are not small. The first moment of the distribution is as large as 218. This indicates that only part of the coordinate change can be explained by collective motion of an ANM. Nevertheless, the 50 lowest normal modes cover about 50 % of the coordinate change. Similar tendencies were observed for the other structural transitions discussed in this work (data not shown).

Based on our definition of fractional coverage *f*_1_ of the conformational change by a small basis of slow normal modes (see Sect. “Methods”), we have tested to what extent structural transitions can be described by a reduced basis of 50 normal modes, corresponding to about 5 % of the total number of modes for the core of transporters with the LeuT fold. This number of modes is a compromise between precision of the description and the effort required for obtaining distance constraints in order to perform such fits with the algorithm described in (Zheng and Brooks 2006).

The results are compiled in Table 3 for all transitions with significant motion of core TMDs. In most cases, 45–50 % of the structural change can be reproduced with the lowest 50 modes. However, only 26 % of the change is covered for the Ce (3LRB) → CSe (3OB6) transition in AdiC and only 36 % for the CSic (3DH4) → Ci (2XQ2) transition in vSGLT. Unsurprisingly, the ANMs fare worse when the structural change is mainly caused by substrate binding, which is dominated by formation of specific interactions that are ignored in the ANMs. Substrate binding to Mhp1 from the outside (2JLN → 2JLO) appears to be an exception. We may not exclude that this exception arises from only partial remodeling of the structure for those parts of the electron density that exhibit the strongest differences. Recomputation of the normal modes does not improve coverage for Δ_exp_ < 1.5 Å, but does so for larger structural changes.

**Table 3:** Coverage of coordinate changes during structural transitions by the 50 lowest normal modes of ANMs

Transition	*b* (%)^a^	Δ_exp_ (Å)^b^	Δ_*B*,0_ (Å)^c^	*f*_0_ (%)^d^	Δ_*B*,1_ (Å)^e^	*f*_1_ (%)^f^
2JLN → 2JLO	4.8	1.23	0.63	48.9	0.66	46.6
2JLN → 2X79	4.8	3.22	2.00	37.9	1.63	49.3
3F3A → 2A65	4.1	1.21	0.66	45.3	0.65	46.5
3LRB → 3OB6	5.0	1.75	1.39	20.8	1.29	26.4
3OB6 → 3L1L	4.8	2.32	1.39	40.0	1.28	44.7
3DH4 → 2XQ2	4.4	1.18	0.76	35.3	0.75	36.3

^a^Percentage of all modes that is contributed by the basis of 50 modes

^b^C^α^ r.m.s.d. between the two structures

^c^C^α^ r.m.s.d. covered by 50 normal modes of the ANM of the initial structure

^d^Fractional coverage by 50 normal modes of the ANM of the initial structure

^e^C^α^ r.m.s.d. covered by 50 iteratively recomputed normal modes

^f^Fractional coverage by 50 iteratively recomputed normal modes

**Fig. 7:**
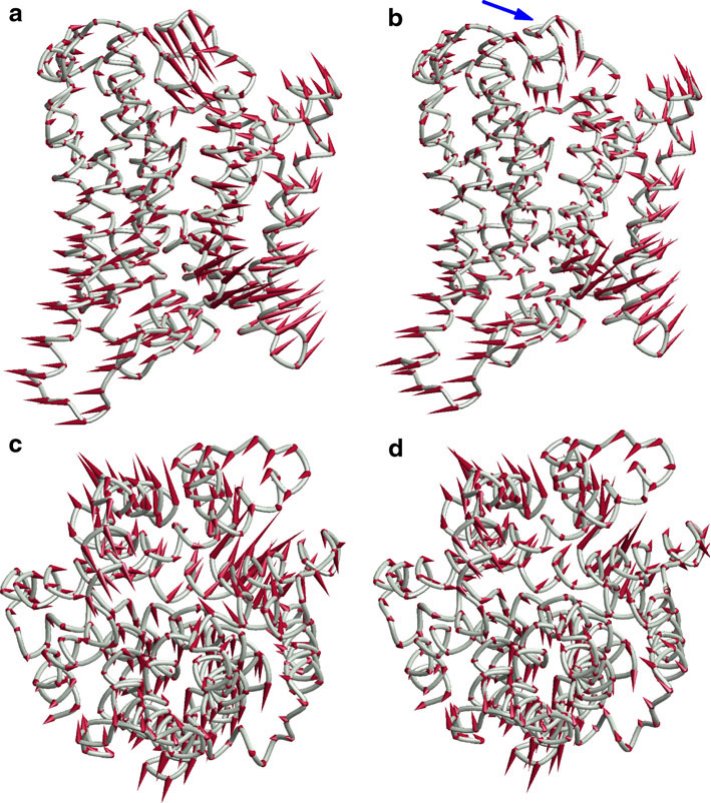
Visualization of the CSec → Ci transition of Mhp1 from the crystal structures of the starting and end conformation (*left*) and from the crystal structure of the starting conformation and the ANM fit result ***R***_f_ for the end conformation (*left*). **a** View parallel to the membrane (periplasmic side up) of a coil model of structure 2JLO (CSec conformation) with motion cones pointing to C^α^ atom locations in structure 2X79 (Ci conformation). **b** View parallel to the membrane of a coil model of structure 2JLO with motion cones pointing to C^α^ atom locations in the ANM fit result ***R***_f_. **c** View normal to the membrane from the periplasmic side of a coil model of structure 2JLO with motion cones pointing to C^α^ atom locations in structure 2X79. **d** View normal to the membrane of a coil model of structure 2JLO with motion cones pointing to C^α^ atom locations in the ANM fit result ***R***_f_

The CSec (2JLO) → Ci (2X79) transition of Mhp1 is visualized in Fig. 7a, c based on the two crystal structures and in Fig. 7b, d based on the crystal structure of the starting conformation 2JLO and the ANM coordinate set ***R***_f_ for the end point. Note that this comparison shows how well the ANM could potentially reproduce the conformational change if driven by a sufficient number of experimental distance constraints. At 49 % coverage of the coordinate change, the gist of the structural transformation is well captured. Apart from moderate errors in direction and amplitude of some of the motion cones, the main deficiency lies in a significant underestimate of the inward motion of L5–6 (blue arrow in Fig. 7b) and a correlated reorientation of TMD 5. Note that the conformational change of L5–6 might indeed be uncoupled from TMD motion. This appears to be feasible since loop conformations differ without an accompanying difference of TMD coordinates in several pairs of crystal structures.

The extent of coverage of the structural transition by the reduced ANM can also be assessed by the characteristic angles. With the ANM model, θ_*B*,4_ decreases by 14.3°, whereas the decrease is 17.1° for the crystal structure 2X79. Hence, the ANM covers most of the motion of the hash relative to the bundle. Of the increase in ϕ_10_ by 17.6°, the ANM covers 10.4° and of the decrease in ϕ_5_ by 24.1° it covers 11.9°. Again unsurprisingly, the more collective motion of the hash with respect to the bundle is better reproduced than the less collective motion of the arms. Note however that the increase of 18.9° in ϕ_10_ for the Ce (2JLO) → CSec (2JLN) transition of Mhp1 is well covered (17°). In this case the structural change is confined to TMD 10 and L9–10, and 50 normal modes are apparently sufficient to reproduce this less complex motion.

**Fig. 8:**
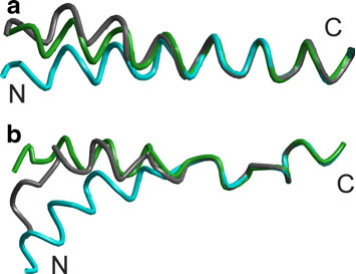
Coverage of TMD internal conformation changes during the CSe (3OB6) → CSec (3L1L) transition of AdiC. *Color code* Crystal structure 3OB6 *cyan*, crystal structure 3L1L *green*, ANM fit result ***R***_f_ *grey*. **a** TMD 2. **b** TMD 6

We have also tested to which extent internal conformation changes of TMDs are reproduced by ANM fitting with 5 % of the normal modes. The most pronounced changes are observed for the CSe (3OB6) → CSec (3L1L) transition of AdiC, in particular for TMDs 2 and 6. As can be seen in Fig. 8 again the gist of these changes is captured by ANM fitting with a reduced basis. For TMD 2 the fit slightly overestimates the change. The N terminal end of TMD 6 unwinds in the ANM fit, probably owing to problems in covering the conformational change of L5–6.

All these findings combined suggest that the lowest 5 % of the normal modes of ANMs can provide a reasonable, coarse description of large-scale structural changes in secondary transporters with the LeuT fold. This approach has an inherent bias to underestimate the amplitude of structural changes. Interpretation of the results at the residue level should be avoided.

### Large-scale structural changes during secondary active transport

Coarse-grained analysis of the structural changes in terms of TMD axes movements (angles θ_*B*,4_, ϕ_5_, and ϕ_10_) and analysis of the per-residue covariance matrix of ANM strongly suggest that the relative motion of the bundle of TMDs 1, 2, 6, and 7 with respect to the hash motif of TMDs 3, 4, 8, and 9 is a general feature of transporters with the LeuT fold. In particular, angle θ_*B*,4_ between the mean bundle axis and the mean axis of the invariably straight TMD 4 in the hash motif is strongly correlated with the conformational states of the proteins. This angle decreases by about 20° during the transition from outward-open to inward-open states. The correlation of changes in angles ϕ_5_ and ϕ_10_ with the conformational states indicates that arms I (TMD 5) and II (TMD 10) are generally involved in occlusion of the cytoplasmic and periplasmic pathway to the central binding site, respectively. These findings support and extend the rocking-bundle model for the major structural transition in these proteins, which was originally suggested in (Forrest et al. 2008) and extended in (Shimamura et al. 2010).

We also find that the bundle and hash do not strictly move as rigid bodies. In almost all structural changes at least one TMD slightly reorients with respect to the other TMDs in either hash or bundle, and quite often, TMDs slightly flex to occlude or open pathways to the central binding site, in particular, TMDs 1, 2, 8, and 10. These findings qualitatively agree with observations made on unbiased MD simulation trajectories for a homology model of the human serotonin transporter SERT (Koldsø et al. 2011). They are also in line with the ANM covariance matrices, which indicate differences in coupling strength between different pairs of TMDs within the hash and bundle motif. In the amino acid antiporter AdiC, TMD 6 undergoes a large-scale change of its internal conformation to occlude the periplasmic pathway.

## Conclusion

Superposition of the ten-helix cores of secondary transporter structures in the LeuT fold reveals that relative arrangement of the TMDs is dominated by the functional state, i.e., outward- or inward-open conformation and presence or absence of occlusion of the cytoplasmic and periplasmic path to the central binding site rather than by peculiarities of the individual proteins. Three angles that characterize this relative arrangement correlate well with the functional states. This correlation and analysis of transitions between crystal structures of the same protein for Mhp1, LeuT, AdiC, and vSGLT support the rocking bundle model, which stipulates that the major conformational change in the outward-open to inward-open transition is the relative motion of the bundle of core TMDs 1, 2, 6, and 7 with respect to the hash motif consisting of TMDs 3, 4, 8, and 9. Furthermore, the arm TMDs 5 and 10 appear to play an important role in occlusion of the cytoplasmic and periplasmic pathways to the central binding site. Such occlusion is further aided by slight reorientation of TMDs within the hash and bundle, and by slight internal conformation changes of kinked TMDs and moderate internal conformation changes of partially unwound TMDs.

This picture of relative TMD motion is in qualitative agreement with the coupling between TMDs suggested by the per-residue covariance matrix of anisotropic elastic network models, which exhibits similar features for all known structures in the LeuT fold. The observed relative motions of the TMDs and, to some extent, of the intervening loops can be qualitatively reproduced by the 5 % lowest frequency normal modes of the network models, although these modes cover slightly less than 50 % of the coordinate change. The slow normal modes provide only a poor description for conformational changes that are dominated by substrate binding.

These results set the stage for testing hypotheses on structural transitions by EPR distance measurements between spin labels and for modeling the changes by constraint-based fitting of anisotropic elastic network models.

## Electronic supplementary material

Below is the link to the electronic supplementary material.

**Online resource 1**: PDF file with figures that visualize structural transitions of the ten-helix core in the LeuT fold. Each page shows a coil model of the starting structure with color coding of the TMDs as in Fig. 1. Views are along the membrane plane with the periplasmic side on top (top left) and perpendicular to the membrane plane from the periplasmic side (center left). The right column shows the same coil model along the same viewing directions without color coding with added crimson motion cones pointing towards the C^α^ atom locations in the end structure of the transition. The bottom left shows a color-coded plot of the C^α^ atom displacements versus residue number starting with the first residue of the core. Consult Table 1 to convert to residue numbers in the crystal structure. (PDF 11284 kb)
