# Supplementary material for: A comparative study of structures and structural transitions of secondary transporters with the LeuT fold
Source: Eur Biophys J. 2012 May 3;42(2):181–97. doi: 10.1007/s00249-012-0802-z (PMC3578728; doi:10.1007/s00249-012-0802-z)

A comparative study of structures and structural transitions of secondary transporters with the LeuT fold

- Online Resource -

G. Jeschke

Lab. Phys. Chem., ETH Zürich, Wolfgang-Pauli-Strasse 10,
CH-8093 Zürich, Switzerland

gjeschke@ethz.ch

Visualization of structural transitions. Each page shows in the left column the ten-helix core with color coding as in Figure 1 of the paper with viewing directions along the lipid bilayer plane (top) and along the bilayer normal (middle). The right column visualizes the transition with the initial structure shown as a grey coil model and crimson cones pointing to C^{α} atom coordinates in the final structure.The bottom plot in the left column shows the per residue coordinate shift of the C^{α} atoms in units of Å.

2JLN → 2JLO

Ce → CSec

Mhp1

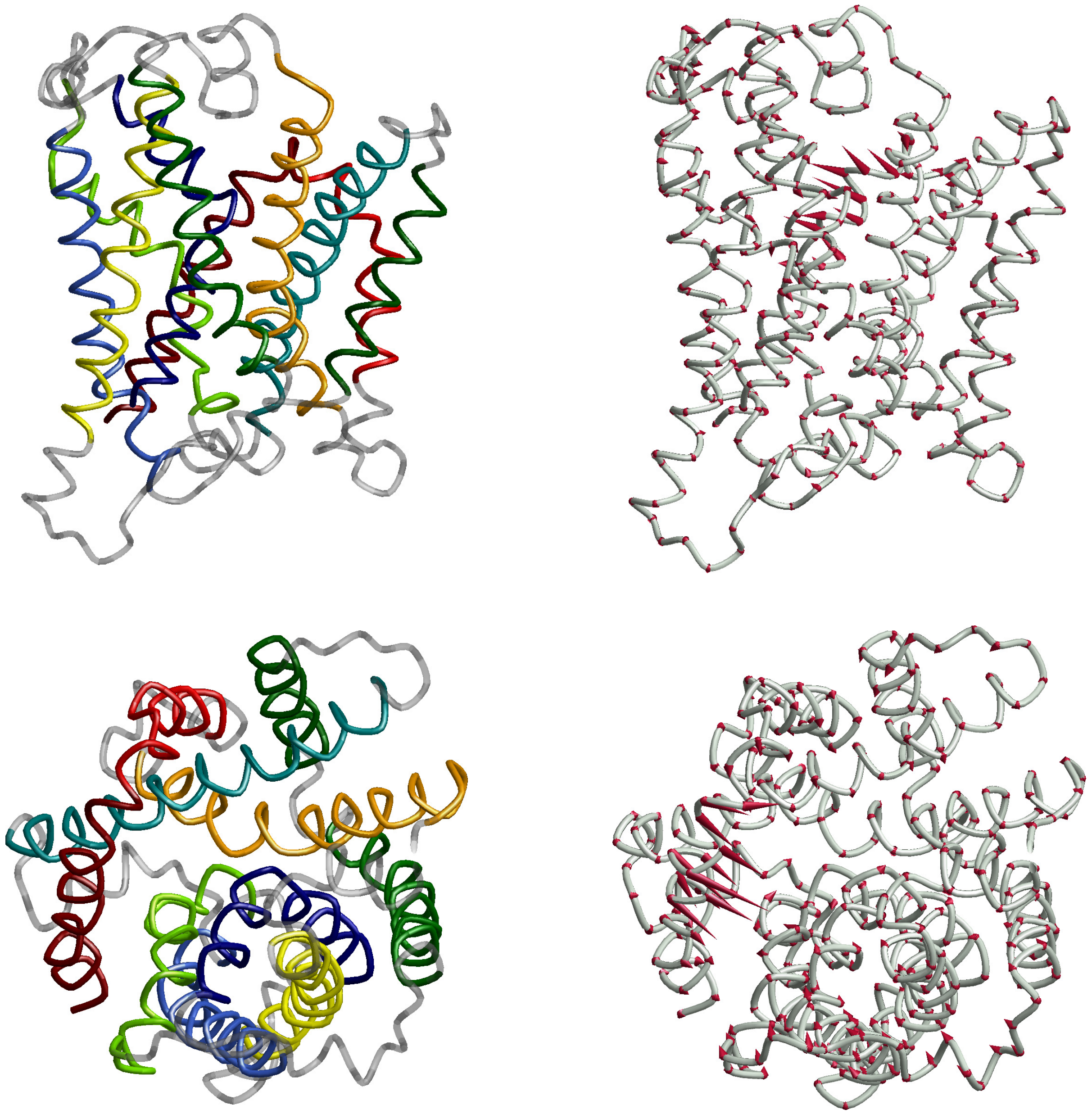

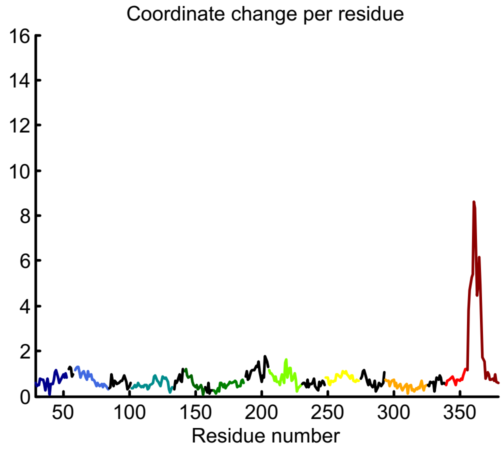

2JLO → 2X79

CSe → Ci

Mhp1

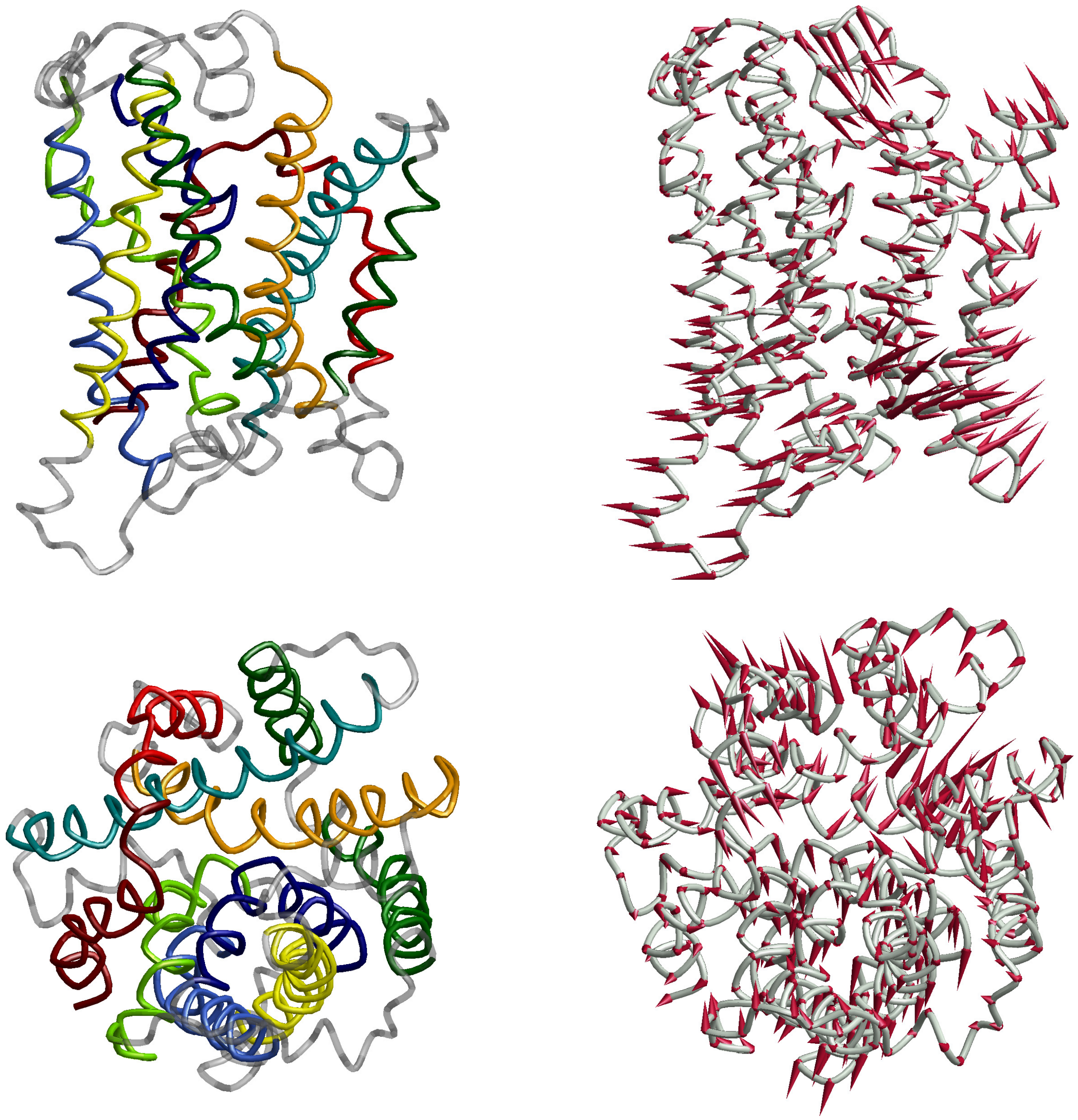

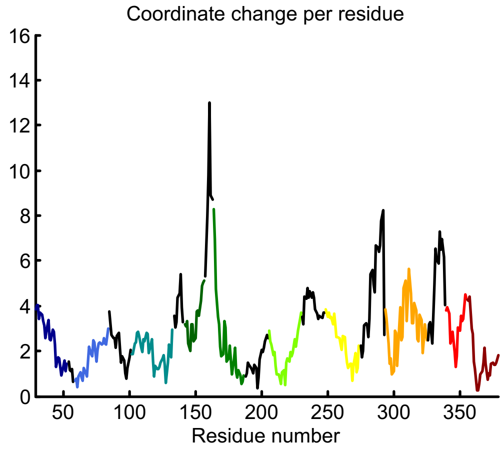

3F3A → 2A65

CSe → CSec

LeuT

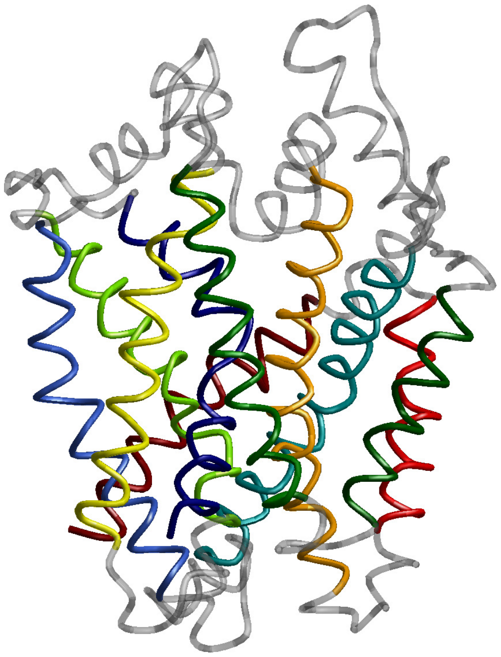

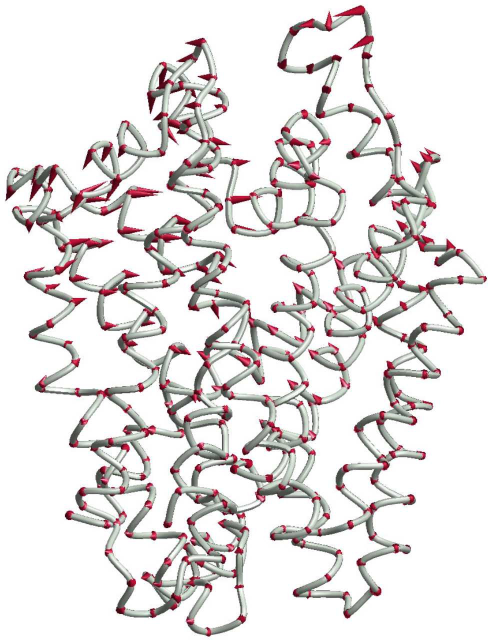

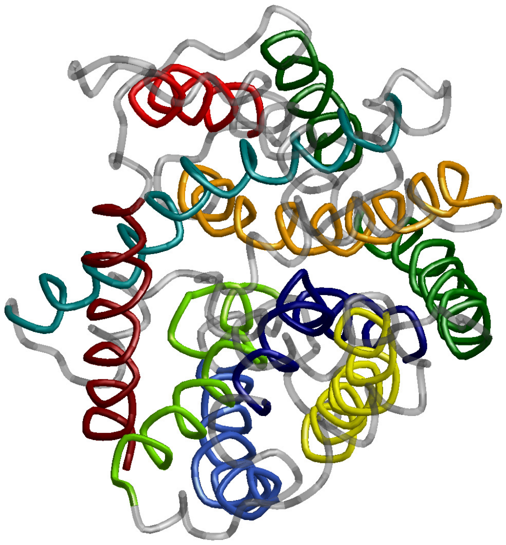

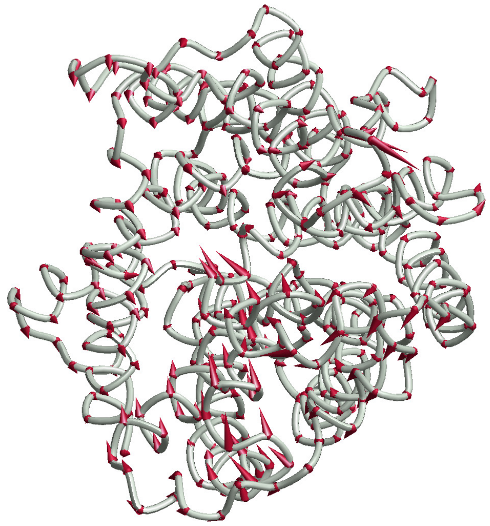

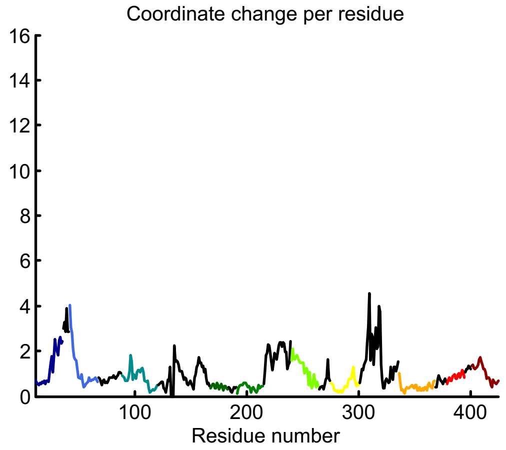

2A65 → 3GJC

CSec → S2

LeuT

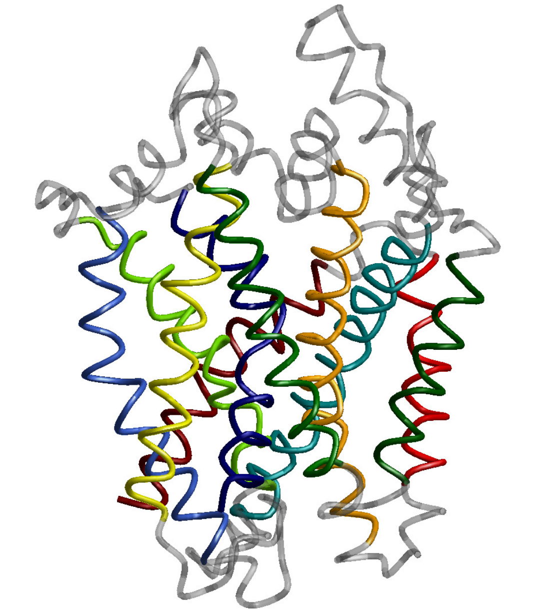

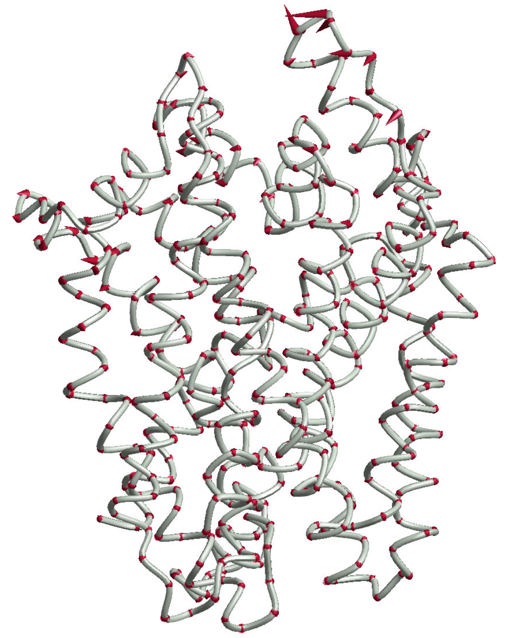

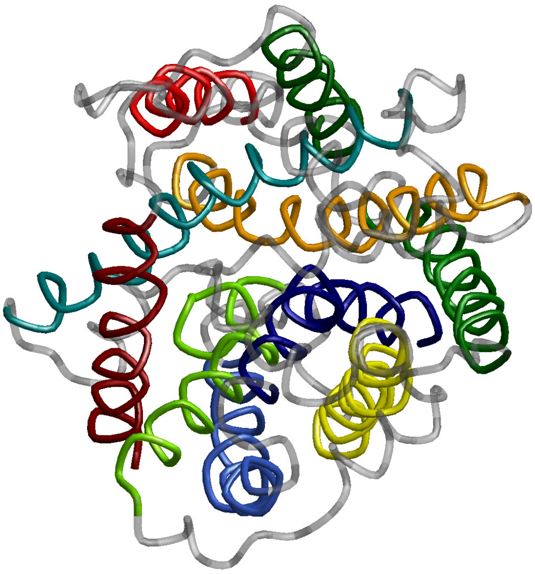

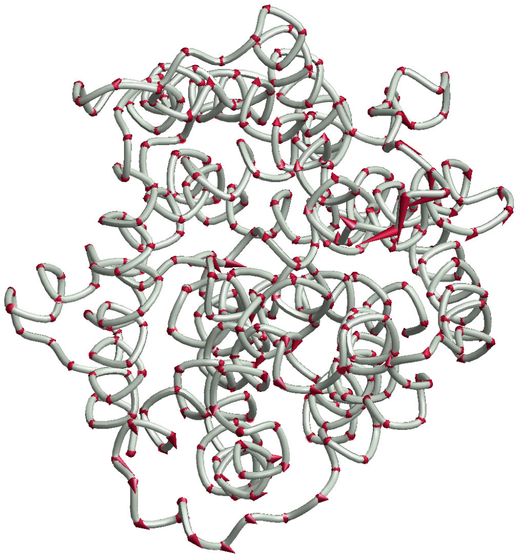

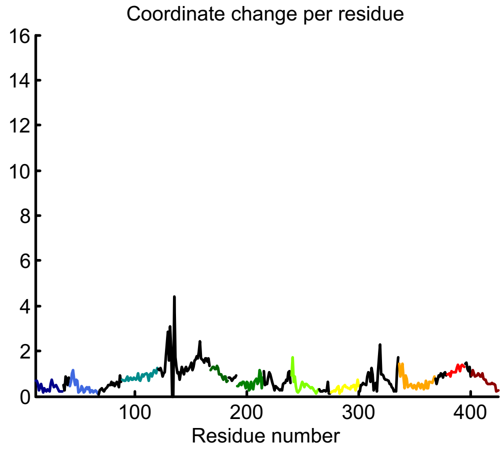

2A65 → 3TT3

CSec → Ci

LeuT

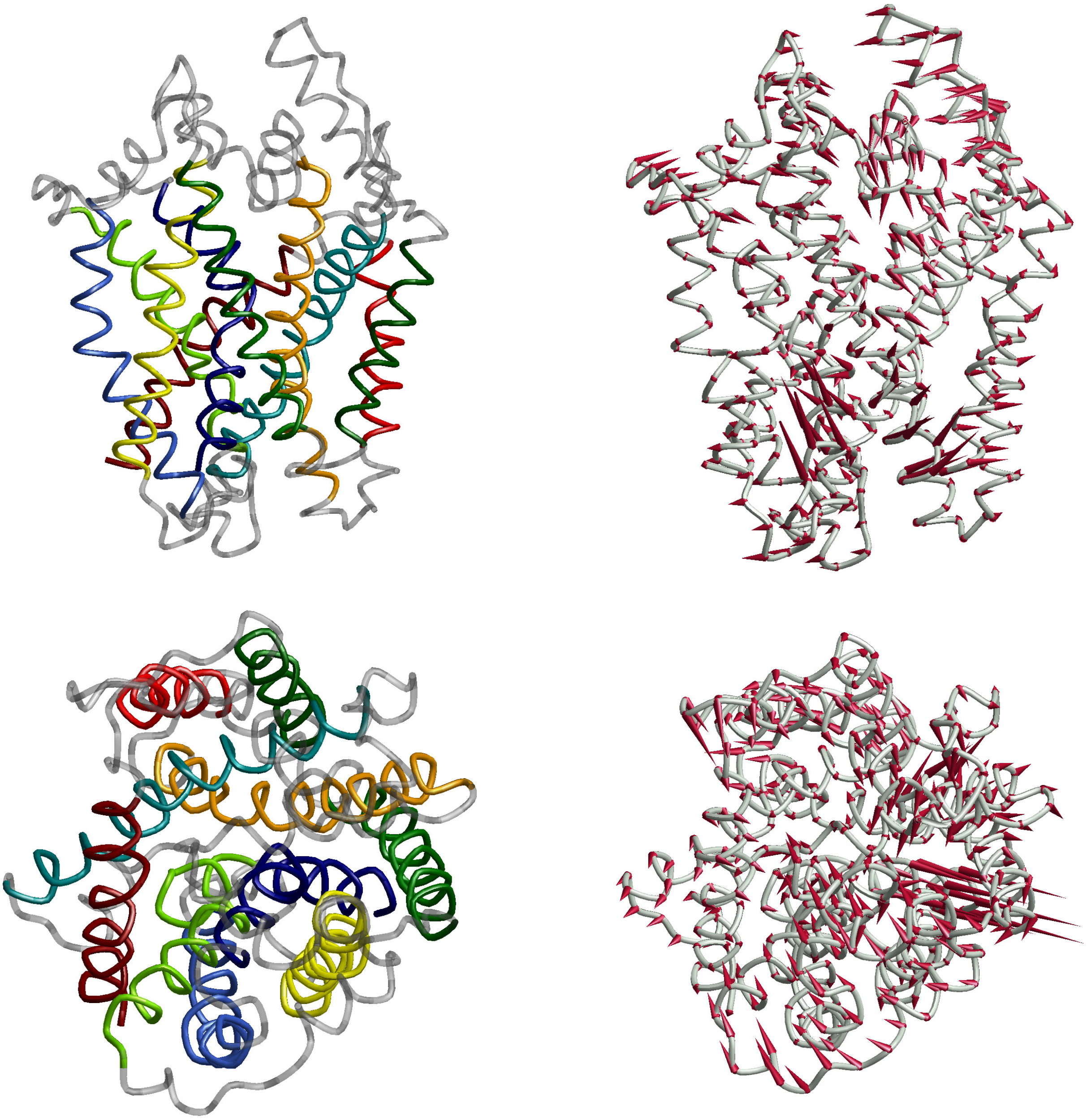

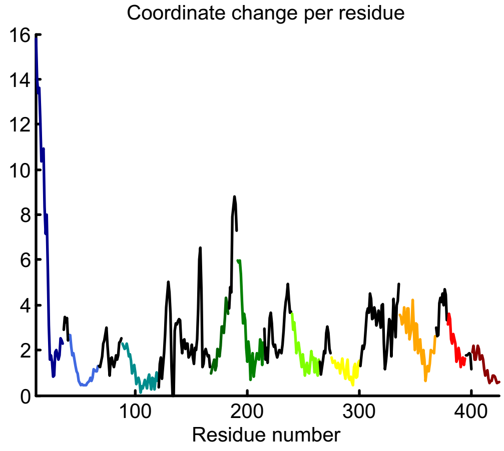

3LRB → 3OB6

Ce → CSe

AdiC

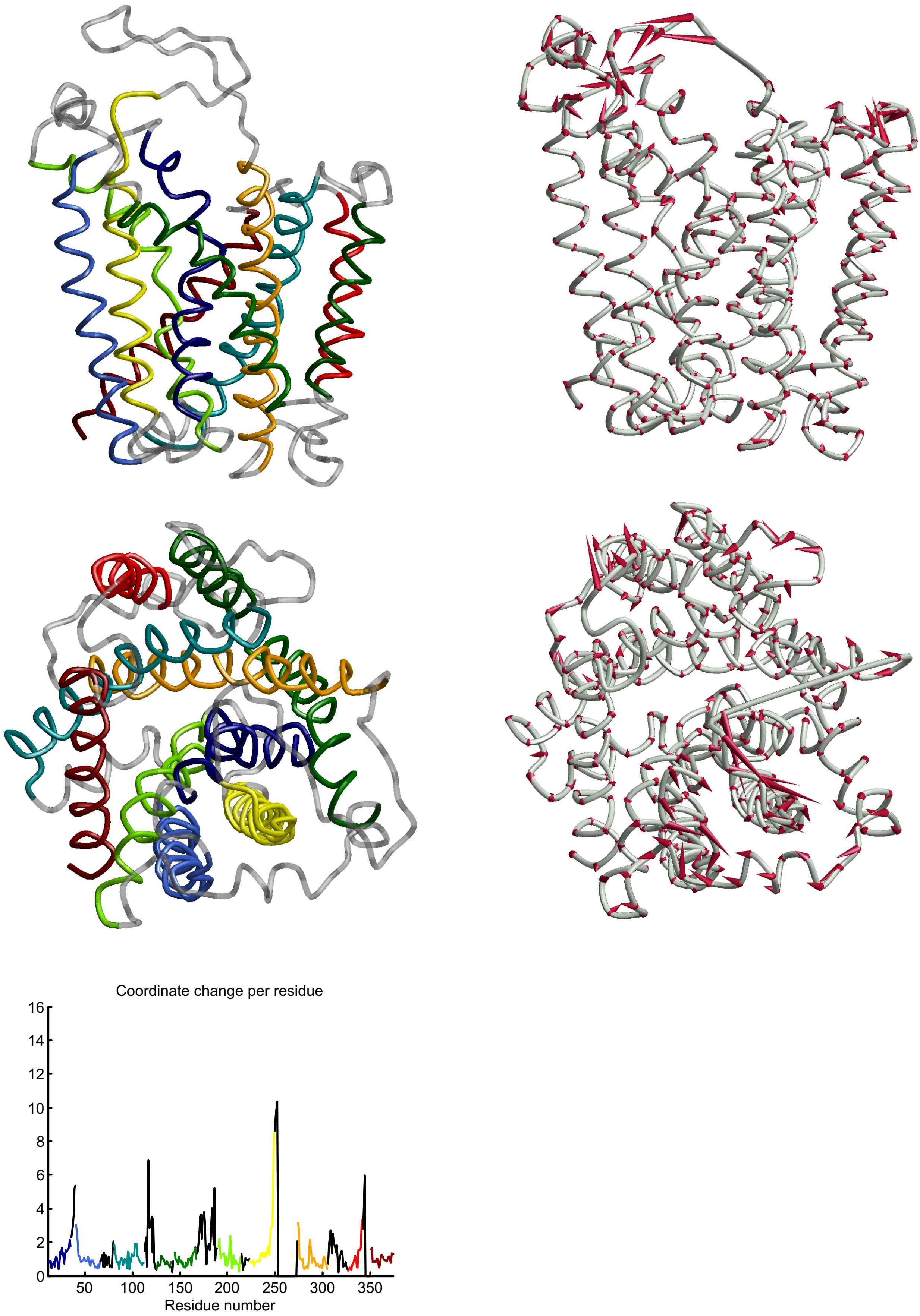

3OB6 → 3L1L

CSe → CSec

AdiC

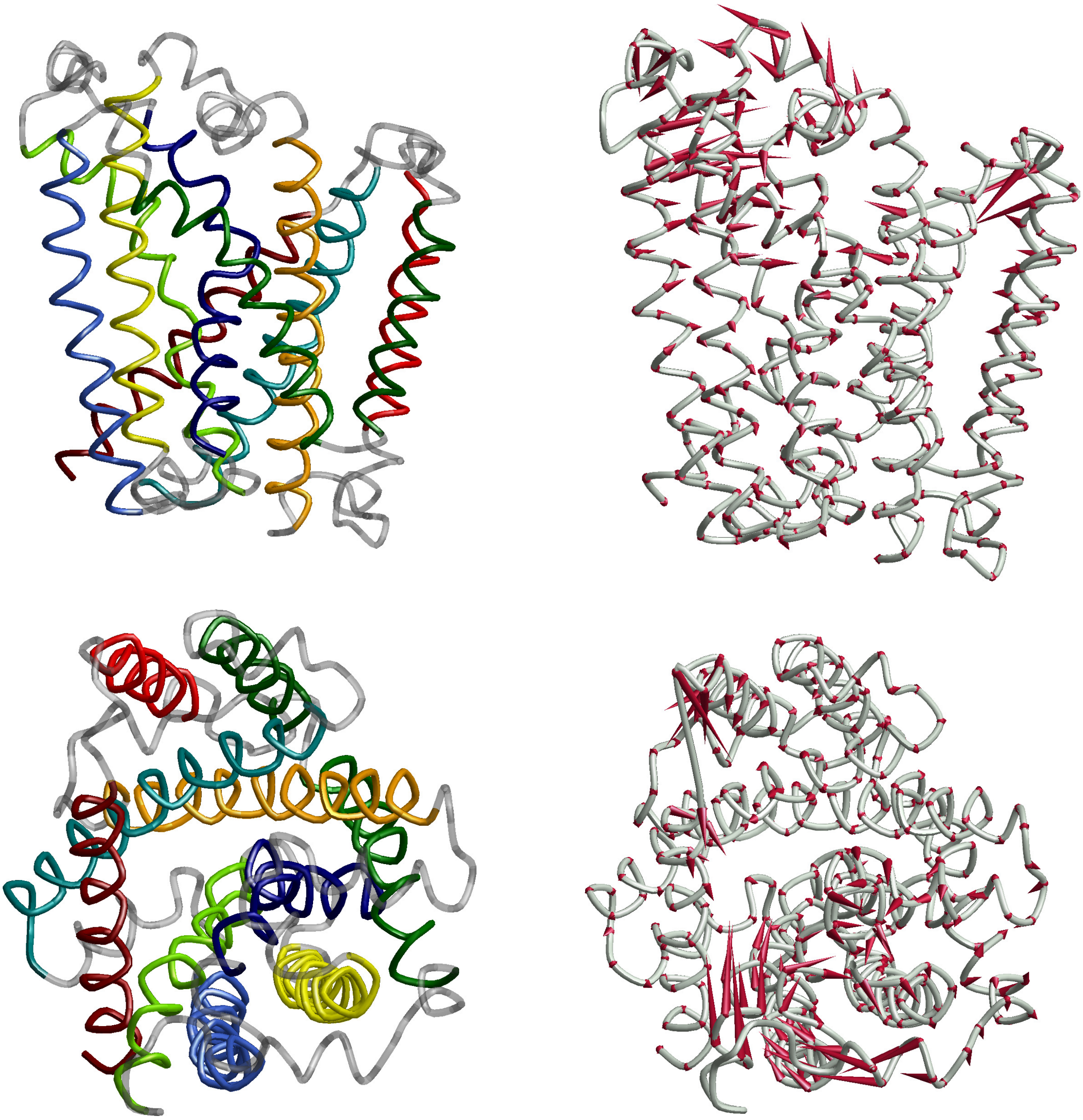

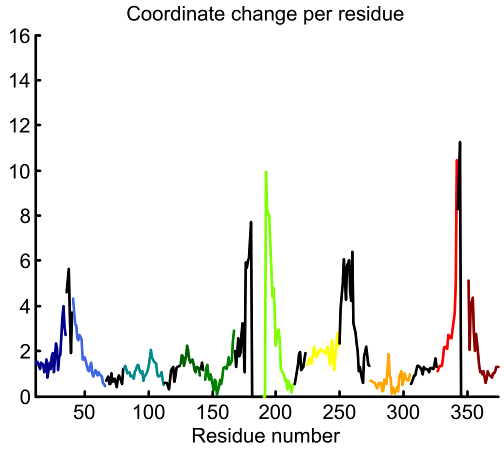

3LRB → 3NCY

Ce → Ce

AdiC

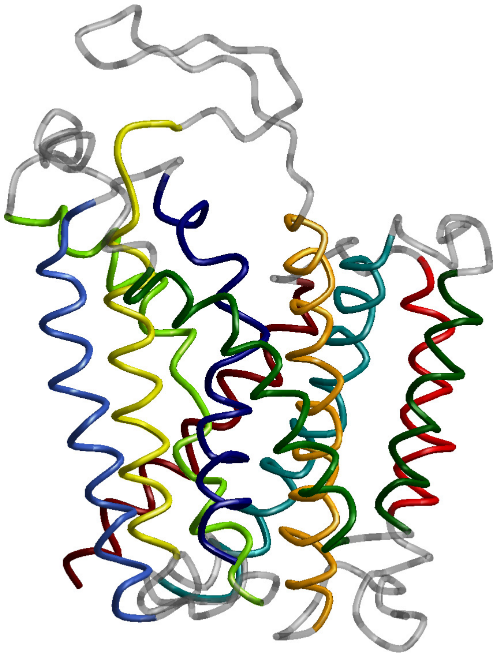

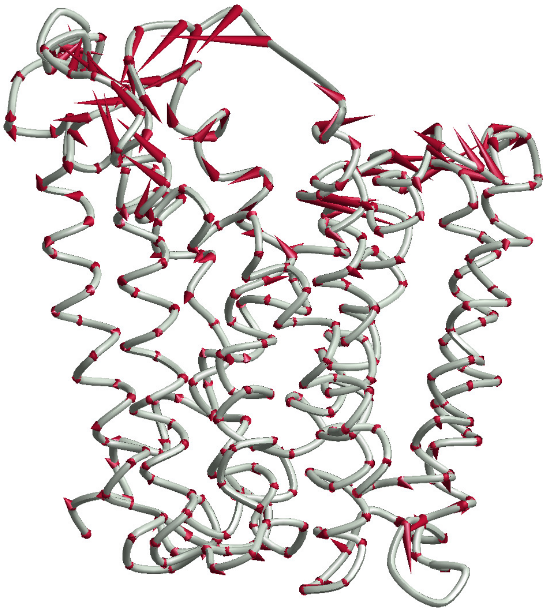

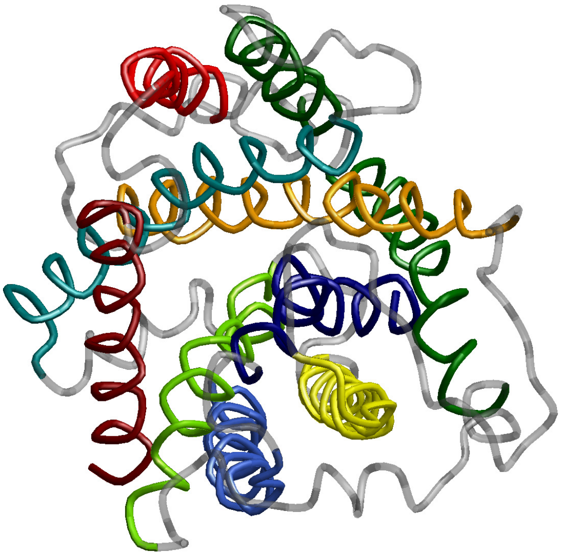

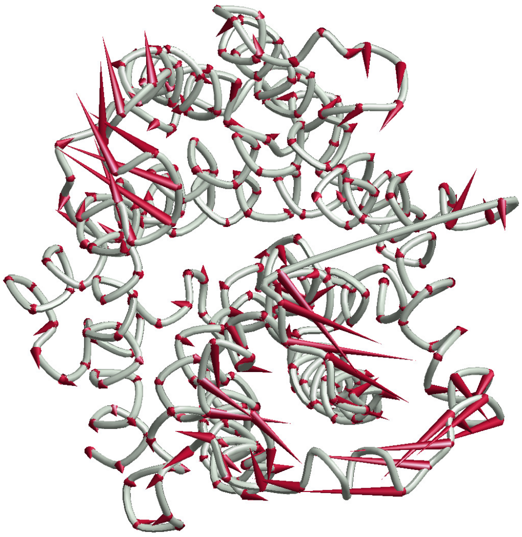

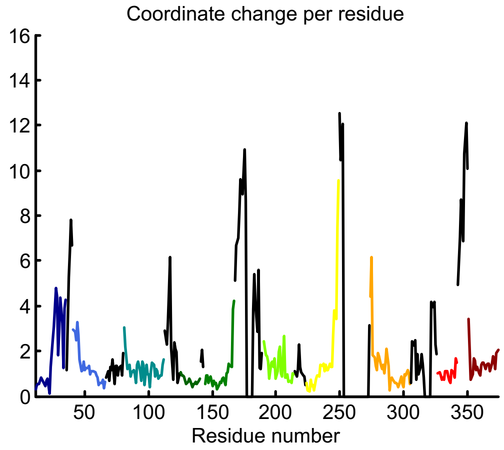

3DH4 → 2XQ2

CSic → Ci

vSGLT

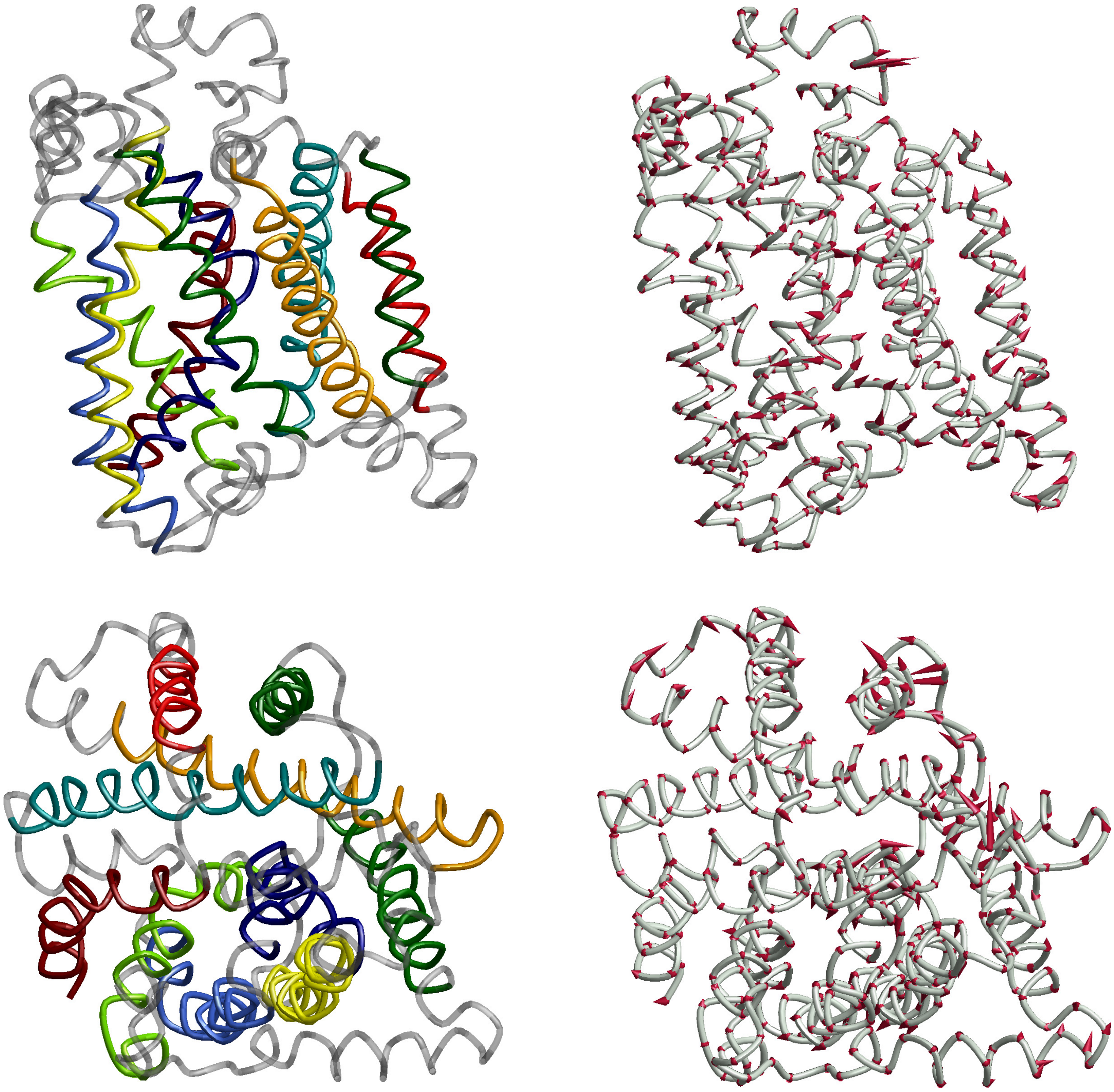

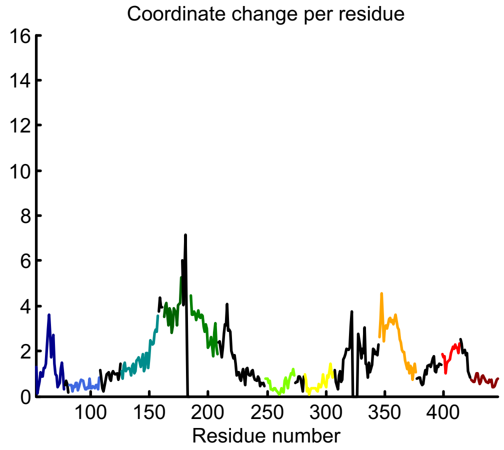

Supplement: Supplementary file 1 — Online resource 1: PDF file with figures that visualize structural transitions of the ten-helix core in the LeuT fold. Each page shows a coil model of the starting structure with color coding of the TMDs as in Fig. 1. Views are along the membrane plane with the periplasmic side on top (top left) and perpendicular to the membrane plane from the periplasmic side (center left). The right column shows the same coil model along the same viewing directions without color coding with added crimson motion cones pointing towards the Cα atom locations in the end structure of the transition. The bottom left shows a color-coded plot of the Cα atom displacements versus residue number starting with the first residue of the core. Consult Table 1 to convert to residue numbers in the crystal structure. (PDF 11284 kb) [file 249_2012_802_MOESM1_ESM.pdf]
